# Comprehensive transcriptomic characterization reveals core genes and module associated with immunological changes via 1619 samples of brain glioma

**DOI:** 10.1038/s41419-021-04427-8

**Published:** 2021-12-08

**Authors:** Ying Zhang, Wenping Ma, Wenhua Fan, Changyuan Ren, Jianbao Xu, Fan Zeng, Zhaoshi Bao, Tao Jiang, Zheng Zhao

**Affiliations:** 1 Beijing Neurosurgical Institute, Capital Medical University, 100070 Beijing, China; 2 Chinese Glioma Genome Atlas Network (CGGA) and Asian Glioma Genome Atlas Network (AGGA), 100070 Beijing, China; 3 Department of Neurosurgery, Beijing Tiantan Hospital, Capital Medical University, 100070 Beijing, China; 4 Sanbo Brain Hospital, Capital Medical University, 100093 Beijing, China; 5 The Second Affiliated Hospital of Harbin Medical University, 150001 Harbin, China; 6 Center of Brain Tumor, Beijing Institute for Brain Disorders, 100069 Beijing, China; 7 China National Clinical Research Center for Neurological Diseases, 100070 Beijing, China

**Keywords:** Tumour immunology, Prognostic markers, CNS cancer

## Abstract

Glioma is the most common primary malignant brain tumor with limited treatment options and poor prognosis. To investigate the potential relationships between transcriptional characteristics and clinical phenotypes, we applied weighted gene co-expression network analysis (WGCNA) to construct a free-scale gene co-expression network yielding four modules in gliomas. Turquoise and yellow modules were positively correlated with the most malignant glioma subtype (*IDH*-wildtype glioblastomas). Of them, genes in turquoise module were mainly involved in immune-related terms and were regulated by *NFKB1*, *RELA*, *SP1*, *STAT1* and *STAT3*. Meanwhile, genes in yellow module mainly participated in cell-cycle and division processes and were regulated by *E2F1*, *TP53*, *E2F4*, *YBX1* and *E2F3*. Furthermore, 14 genes in turquoise module were screened as hub genes. Among them, five prognostic hub genes (*TNFRSF1B*, *LAIR1*, *TYROBP*, *VAMP8*, and *FCGR2A*) were selected to construct a prognostic risk score model via LASSO method. The risk score of this immune-related gene signature is associated with clinical features, malignant phenotype, and somatic alterations. Moreover, this signature showed an accurate prediction of prognosis across different clinical and pathological subgroups in three independent datasets including 1619 samples. Our results showed that the high-risk group was characterized by active immune-related activities while the low-risk group enriched in neurophysiological-related pathway. Importantly, the high-risk score of our immune signature predicts an enrichment of glioma-associated microglia/macrophages and less response to immune checkpoint blockade (ICB) therapy in gliomas. This study not only provides new insights into the molecular pathogenesis of glioma, but may also help optimize the immunotherapies for glioma patients.

## Introduction

Gliomas are tumors that arise from glial or precursor cells, which accounts for 80% of malignant tumors in central nervous system (CNS) [1]. With surgical resection, radiotherapy, and chemotherapy treatment, the patient’s prognosis remains dismal [2]. In 2016 CNS WHO classification, gliomas are further classified by histological and molecular characteristics, including lower-grade gliomas (LGG), isocitrate dehydrogenase-mutant (*IDH*-mutant) and 1p/19q-codeleted; LGG, *IDH*-mutant without 1p/19q-codeleted; LGG, *IDH*-wildtype; glioblastomas (GBM), *IDH*-mutant; and GBM, *IDH*-wildtype [3]. Moreover, a series of key genetic alterations are reported in gliomas, including *MGMT* promoter methylation, *EGFR* amplification, and *MET* gene fusion [4, 5]. The diversity of genetic alterations and gene expression changes contribute to high heterogeneity and resistance to treatment of gliomas.

Tumor microenvironment (TME) plays a pivotal role in the occurrence and development of tumors. In addition to cancer cells, TME is formed by many non-malignant cells, including immune cells (macrophages, microglia, T lymphocytes), endothelial cells, fibroblasts, and others [6, 7]. Nowadays, many computational methods have been developed to estimate the types and fraction of cells in tumor samples based on expression data. These provide a landscape of TME to facilitate the understanding of tumor progression and the design of new efficient immune therapies.

Glioma-associated microglia/macrophages (GAMs) are the most multifunctional cells in glioma TME, accounting for approximately 30–50% of the total cell population [8]. Depending on the respective stimuli, the polarization of microglia/macrophages is either towards the pro-inflammatory/anti-tumor phenotype or towards the anti-inflammatory/pro-tumorigenic phenotype [9]. In glioma, GAMs are more likely to possess anti-inflammatory and pro-tumorigenic phenotype [10], which enhances glioma invasion, angiogenesis, tumor growth and contributes to an immunosuppressive TME [11].

In this study, we utilized the weighted gene co-expression network analysis (WGCNA) to construct a free-scale gene co-expression network related to glioma patients’ clinical molecular pathological traits. Then, we constructed and validated an immune-related prognostic model using 1619 glioma cases from three datasets. Furthermore, we explored the detailed relationship between the risk score model and the landscape of immune-related profiles and response to ICB therapy, especially anti-PD-1 immunotherapy. Our findings provide a powerful prognostic tool for glioma patients, give new insights into the molecular pathogenesis of glioma, and further may help optimize immunotherapies for glioma patients.

## Materials and methods

### Sample selection, data processing, and study design

Transcriptional RNA sequencing data, whole-exome sequencing data, and corresponding clinical traits information were downloaded from the Chinese Glioma Genome Atlas database (CGGA, 325 samples for mRNAseq_325 dataset; 693 samples for mRNAseq_693 dataset; 286 samples for WEseq_286; http://www.cgga.org.cn) [12, 13]. The patients in CGGA datasets are Chinese people. CGGA325 and CGGA693 are two independent cohorts that are collected by the same team at different times. RNA sequencing data (702 samples), somatic mutation, and copy number alterations (CNAs) data (646 samples), and corresponding clinical traits information were obtained from The Cancer Genome Atlas (TCGA, https://portal.gdc.cancer.gov/) as a validation dataset, which are mainly Caucasian. According to the WHO 2016 classification criterion, these samples were classified into five subtypes (*IDH*-mutant with chromosome 1p/19q codeletion LGG; *IDH*-mutant without chromosome 1p/19q codeletion LGG; *IDH*-wildtype LGG; *IDH*-mutant GBM; *IDH*-wildtype GBM). The flowchart is shown in Supplementary Fig. S1.

### Gene co-expression network construction and identification of candidate hub genes

The ‘WGCNA’ package in R language was performed to construct a co-expression network of the top 5000 most variant genes, which were calculated by a robust method called median absolute deviation (MAD) [14]. First, a matrix of adjacencies was built according to the Pearson’s correlation value between paired genes. Then, we chose the soft thresholding power *β* = 14 to construct an unsigned scale-free co-expression network in this study. The weighted adjacency matrix was transformed into topological overlap matrix (TOM). Thirdly, a standard method, cutreeDynamic function, was performed to identify co-expression gene modules with a module minimum size of 30 and merge height cut of 0.25. The dissimilarity of the module eigengenes (MEs) was calculated by the moduleEigengenes function in the ‘WGCNA’ package. The association between MEs with clinical subtypes of gliomas was assessed by Spearman’s correlation. A *p* value < 0.05 was significant, and the module highly correlated with clinical subtypes of gliomas was selected for further analysis. To identify candidate hub genes for further analysis, we calculated the correlation between individual gene and clinical subtypes of gliomas (Gene Significance, GS), as well as the correlation between individual genes and MEs (Module Membership, MM). The candidate hub genes were screen out with MM > 0.8 and GS > 0.2 in each target module as threshold.

### Transcriptional regulatory analysis

Transcriptional Regulatory Relationships Unraveled by Sentence-based Text mining (TRRUST, https://www.grnpedia.org/trrust/) was performed to identify transcription regulators (TFs) that significantly targeted candidate hub genes in each module (*FDR* < 0.05) [15]. Then the results were visualized by Cytoscape.

### Protein−protein interaction analysis and identification of hub genes

The connectivity among candidate hub genes in turquoise module was visualized by Cytoscape, and a connectivity weight > 0.2 was set as a threshold. Meanwhile, the protein-protein interaction network of these genes was analyzed by the STRING database (https://string-db.org/) and graphed by Cytoscape. The MCODE plug based on Cytoscape was used to identify core cluster genes in these two networks. Venn analysis was performed to compare these results (http://bioinformatics.psb.ugent.be/webtools/Venn/).

### Functional enrichment analysis

Functional enrichment analysis was performed by Metascape (http://metascape.org) [16]. The protein-protein interaction network was analyzed by the STRING database (https://string-db.org/) and presented by Cytoscape. The software GSEA downloaded from Broad Institute was used for analysis and only gene sets with p.adjust < 0.05 were considered as significant. The results were presented by ‘ggplot2’, ‘enrichplot’, and ‘clusterProfiler’ packages in R.

### Construction of a prognostic risk score model

A univariate Cox proportional regression analysis was performed to calculate the association between the expression of 14 hub genes and overall survival (OS) in the CGGA dataset. Next, we performed the LASSO method and selected five prognostic genes to construct a prognostic risk model. Finally, the following formula was used to calculate the risk score for each patient in CGGA training and TCGA validation datasets.

$${{{\mathrm{Risk score}}}} = \mathop {\sum }\limits_{i = 1}^n {{{\mathrm{expr}}}}_{{{{\mathrm{gene}}}}({{{{i}}}})} \times {{{\mathrm{Coeff}}}}_{{{{\mathrm{gene}}}}({{{{i}}}})}$$

### G-CIMP prediction based on expression data

We used TCGA and CGGA mRNA expression datasets to predict the glioma-CpG island methylator phenotype (G-CIMP) according to previous studies [17, 18].

### Cell-cycle scoring and regression

We performed Seurat to compute cell-cycle phase based on cell-cycle markers expression in the TCGA and CGGA datasets [19]. The chi-square test was performed to calculate the difference between the high-risk and low-risk groups.

### Correlation of tumor metastasis with risk score in glioma

The local invasion and intravasation scores in each glioma sample were quantified by the single-sample gene-set enrichment analysis (ssGSEA) [20]. The Spearman correlation was performed to evaluate the correlation of ssGSEA score and tumor metastasis.

### Cell types enrichment analysis

Estimation of STromal and Immune cells in MAlignant Tumours using Expression data (ESTIMATE) have been described in our previous study [21]. CIBERSORT (http://cibersort.stanford.edu) method was performed to characterize cell composition based on gene expression profiles in previous study [21, 22]. The relationship between risk score and cell infiltration fraction in glioma was analyzed by Spearman correlation analysis and graphed by R package ‘ggplot2’. The correlation between risk score and different factors was calculated by Pearson analysis and presented by R package ‘corrplot’.

### PD-L1 protein and Tumor Immune Dysfunction and Exclusion (TIDE) analysis

The protein expression of PD-L1 was detected by the reverse-phase protein array (RPPA) analysis and obtained from The Cancer Proteome Atlas (TCPA, http://tcpaportal.org). TIDE is a computational method to predict ICB clinical response based on pre-treatment tumor profiles. This model is based on two mechanisms of tumor immune evasion: the induction of T-cell dysfunction in tumors with high infiltration of cytotoxic T lymphocytes (CTL) and the prevention of T-cell infiltration in tumors with low CTL level [23]. The TIDE score and ICB response of patients with glioma in the CGGA dataset were calculated by TIDE web (http://tide.dfci.harvard.edu) after uploading the scaled transcriptome profiles.

### Immunohistochemistry (IHC) staining

We performed IHC in paraffin-embedded samples obtained from the CGGA sample bank. IHC analysis with AIF1 (Proteintech, 10904-1-AP, 1:500), TNF (Abcam, ab270264,1:150), CD163 (Abcam, ab189915, 1:500), and TIM3 (Abcam, ab241332, 1:500) antibodies were conducted according to our previous procedures [24, 25]. The protein expression levels were evaluated independently by two experienced pathologists and the scoring criteria refer to our published article [25].

### Statistical analysis

Student’s *t* test was performed to calculate the significance of differences between patients in two groups. Fisher test was performed to detect the difference of genomic alterations between the high-risk and low-risk groups. Kaplan−Meier survival analysis and the log-rank test were performed to assess the statistical significance between the high-risk and low-risk groups using R language packages (*survival*, *survminer*, and *ggplot2*). The receiver operating characteristic (ROC) curve analysis was used to evaluate the predictive accuracy and sensitivity of our prognostic model within 1-year, 3-year, and 5-year of OS by ‘*pROC*’ package in R. The independent prognostic factors were identified by univariate and multivariate Cox regression analysis, and presented by ‘*forestplot*’ package in R. An individualized prediction model was constructed using R language packages (*survival* and *rms*). *p* < 0.05 was considered statistically significant.

## Results

### Construction of a prognostic immune signature in glioma

**Fig. 1:**
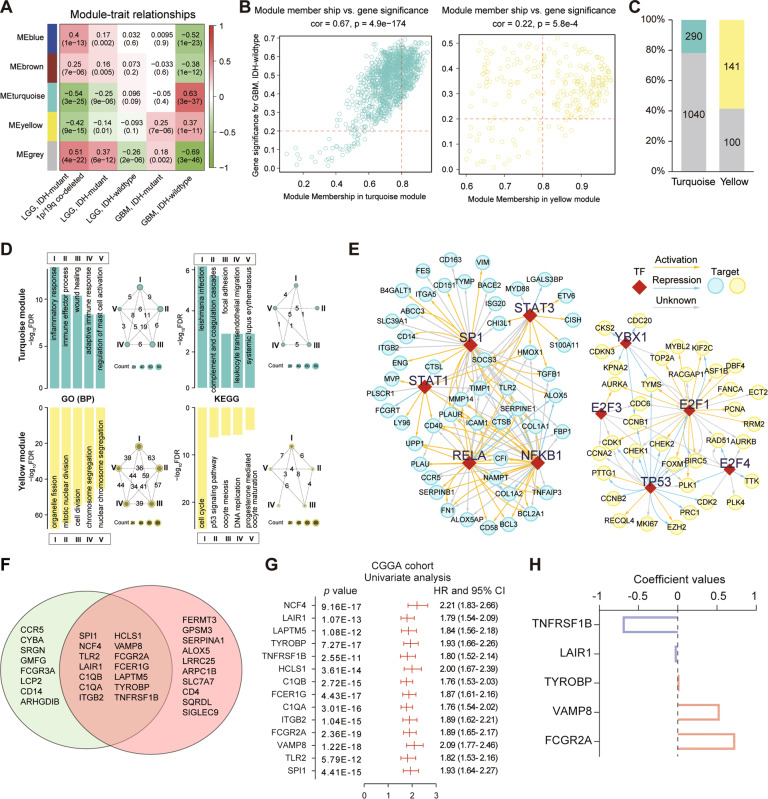
Construction of a prognostic risk signature for OS by WGCNA and LASSO analysis. **A** Heatmap shows correlation between the gene modules and clinical traits. Each cell contains the corresponding correlation and *p* value. **B** The Module Membership (MM) versus Gene Significance (GS) scatterplot for GBM with IDH wildtype in turquoise or yellow module. Each dot represents a gene, and the red lines were set as a threshold for Module Membership > 0.8 and Gene Significance > 0.2. **C** The proportion of candidate hub genes in the turquoise and yellow modules. **D** GO (BP) and KEGG enrichment annotation of candidate hub genes in turquoise or yellow module. GO gene ontology, BP biological process. In the network diagram, the roman number residing in each circle represents a functional term; values on each line indicate number of overlap genes between terms. **E** Key regulated upstream transcription regulators (TFs) of candidate hub genes in module by TRRUST. **F** Hub genes were selected based on overlap between co-expression network and STRING analysis. **G** Univariate Cox regression results for the 14 genes in the CGGA dataset. **H** Coefficient values of the five selected genes by LASSO.

We obtained RNA expression data of 325 glioma samples in CGGA database. First, the genes are ranked according to their variance of gene expression in the samples, and the top 5000 genes were selected for further analysis. We selected *β* = 14 (scale-free *R*^2^ = 0.894) as the soft thresholding power to build a scale-free network (Supplementary Fig. S2A, B). According to their expression pattern, they were divided into four modules by average linkage clustering (Supplementary Fig. S2C, D). After combining clinical traits, we found that *IDH*-wildtype GBM positively correlated with turquoise (*R* = 0.63, *p* = 3e−37) and yellow (*R* = 0.37, *p* = 1e−11) modules, but negatively correlated with blue (*R* = −0.52, *p* = 1e−23) and brown (*R* = −0.38, *p* = 1e−12) modules (Fig. 1A).

According to the threshold (*MM* > 0.8 and *GS* > 0.2), a total of 290 and 141 candidate hub genes were obtained in turquoise and yellow modules, respectively (Fig. 1B, C). Two hundred and ninety candidate hub genes in turquoise module were enriched in ‘*inflammatory response*’, ‘*immune effector process*’, ‘*wound healing*’, and ‘*adaptive immune response*’. The enriched pathways were ‘*complement and coagulation cascades*’, ‘*focal adhesion*’, and ‘*leukocyte transendothelial migration*’ (Fig. 1D). Meanwhile, 141 candidate hub genes in yellow module were enriched in cell-cycle and cell-division related gene ontology (GO) terms. The enriched KEGG pathways were ‘*cell cycle*’, ‘*p53 signaling pathway*’, and ‘*DNA replication*’ (Fig. 1D). These results indicate that these two co-expression modules play distinct roles in the genesis or progression of glioma.

Then, we explored upstream TFs of candidate hub genes via the TRRUST database. A total of 50 and 31 TFs were involved in turquoise and yellow modules, respectively (Supplementary Tables S1, S2). Only two TFs (*SP1* and *PTTG1*) overlapped. The top five TFs for yellow module were *E2F1, TP53, E2F4, YBX1*, and *E2F3*. These targets included *CCNB1, CDK1, CHEK1, CCNA2*, and *CDC6*, which are important cell-cycle and cell division genes (Fig. 1E). Meanwhile, the top five TFs for turquoise module were *NFKB1, RELA, SP1, STAT1*, and *STAT3*, which are regulators of known immune-related genes, such as *TLR2, CD14, CD58, TGFB1* (Fig. 1E). These results indicate that two co-expression modules regulated by different TFs in glioma.

Due to the highest correlation between turquoise module and GBM with *IDH* wildtype, we displayed a highly connected network in this module based on gene co-expression in gliomas. A cut-off of reliability above 0.2 was applied to identify central nodes, and a gene co-expression network complied with 66 nodes and 628 edges was built up and visualized via Cytoscape (Supplementary Fig. S3A). A highly interconnected subnetwork with 24 nodes and 260 edges was identified by MCODE [26] (Supplementary Fig. S3B). Then, the connectivity of these 66 genes was revalidated by STRING database, which supplied functional and physical protein associations (Supplementary Fig. S3C), and three clusters have obtained from this network by MCODE (Supplementary Fig. S3D). Then, 14 genes were selected in both networks and designated as hub genes by VENN analysis (Fig. 1F). Hub genes were significantly related with ‘*leukocyte mediated immunity*’, ‘*immune system process*’, and ‘*myeloid leukocyte*’ activation by STRING and Metascape (Supplementary Fig. S3E, F).

We identified that 14 hub genes were statistically related with OS of glioma patients in the CGGA dataset (Fig. 1G). Then, LASSO regression algorithm was adopted to construct a prognostic immune signature, including *TNFRSF1B, LAIR1, TYROBP, VAMP8*, and *FCGR2A* (Fig. 1H). In both CGGA and TCGA datasets, these genes were associated with gliomas prognosis (Fig. 1G and Supplementary Fig. S4), which is consistent with previous reports that these genes can overenhance or attenuate tumor growth [27–31].

### Association of the risk score with clinical features and malignant phenotype in glioma

**Fig. 2:**
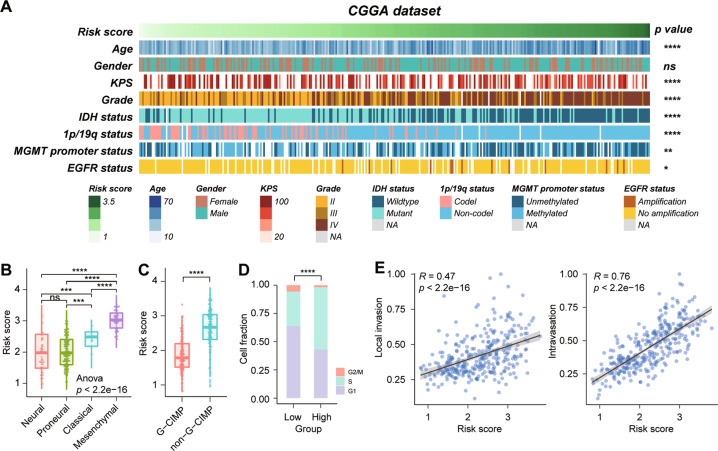
Relationship between the signature risk score and the pathological characteristics in the CGGA dataset. **A** The distribution of clinical and pathological characteristics arranged by the increasing risk score. **B**, **C** Distribution of risk score in patients stratified by TCGA subtype and G-CIMP subtype. **D** The relationship between risk score and cell-cycle proportion in the CGGA dataset. **E** The correlation between risk score and invasion index was analyzed by Pearson correlation analysis. ****p < 0.0001; ***p < 0.001; **p < 0.01; *p < 0.05; n.s. no significance.

According to the risk score, the patients were arranged and the landscape of corresponding pathological characteristic was shown in Fig. 2A. The results indicated that risk scores were significantly higher in elder patients, low Karnofsky Performance Score (KPS), high WHO grade, *IDH* wildtype, without 1p/19q codeletion, *MGMT* promoter unmethylation, *EGFR* amplification patients in the CGGA dataset. Due to larger sample size, a more significant results were shown in the TCGA dataset (Supplementary Fig. S5A). Gain of chr7 and loss of chr10, co-gain of chr19/20 are gathered in patients with high-risk score in the TCGA dataset (Supplementary Fig. S5A). Furthermore, the risk score is higher in mesenchymal subtype (Fig. 2B and Supplementary Fig. S5B) and non-G-CIMP subtype (Fig. 2C and Supplementary Fig. S5C) in the CGGA and TCGA datasets. Compared to the low-risk group, the high-risk group recruited a higher proportion of cases in S (CGGA, Fig. 2D) or G2/M phase (TCGA, Supplementary Fig. S4D), which predict strong cell proliferative activity in the high-risk group. Moreover, the risk score showed a significantly positive correlation with invasion score, especially the intravasation score (Fig. 2E and Supplementary Fig. S5E). Collectively, these results indicate that the risk score of five-gene signature is closely related with the clinical features and malignant phenotype of glioma.

### Somatic variations in the two risk groups

**Fig. 3:**
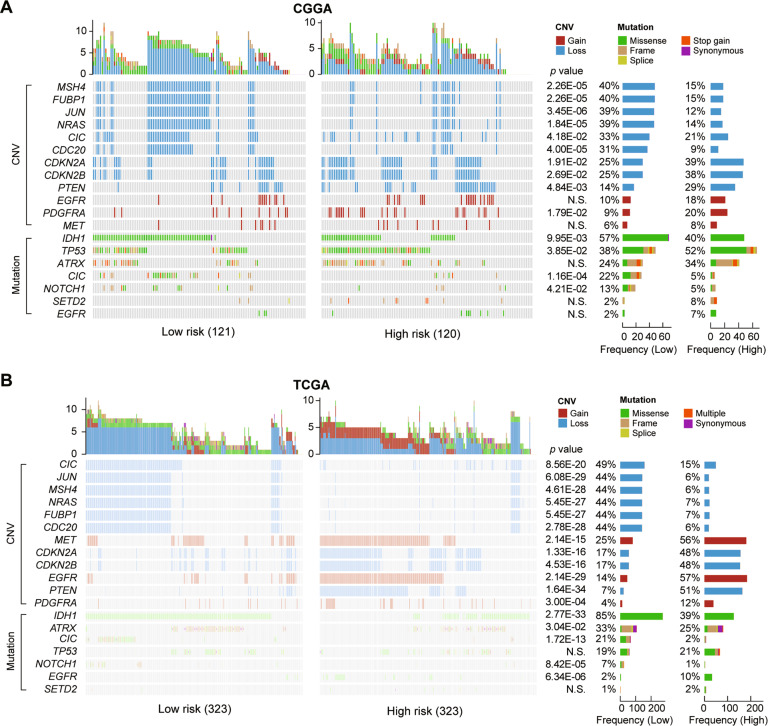
Comparison of genomic alterations between the high-risk and low-risk group in the CGGA and TCGA datasets. Differential somatic mutations and copy number variations analyses within two risk groups (Fisher test) in CGGA (**A**) and TCGA (**B**) datasets. CNV copy number variations; N.S., no significance.

To investigate the difference between the high- and low-risk groups at the genomic level, we analyzed copy number variation (CNV) and somatic mutation from the CGGA and TCGA datasets. Low-risk group had a high frequent deletion in *MSH4*, *FUBP1*, *JUN*, *NRAS*, *CIC*, and *CDC20* in the CGGA dataset (Fig. 3A). In contrast, the high-risk group showed more frequently deleted regions in *CDKN2A*, *CDKN2B*, *PTEN*, and amplification in *PDGFRA*. The low-risk group presented a high mutation frequency in *IDH1*, *CIC*, and *NOTCH1*. While the high-risk group enriched in *TP53* mutation. Furthermore, a more significant result was showed in the TCGA dataset (Fig. 3B), which contained a large sample size. High occurrence of *EGFR* mutation, *EGFR* and *MET* amplification were gathered in patients with high-risk score in the TCGA dataset. *ATRX* mutations are enriched in patients with low-risk score. These findings indicate that gliomas with different risk scores show different genomic alterations.

### The immune-related risk score signature served as an independent prognostic factor

**Fig. 4:**
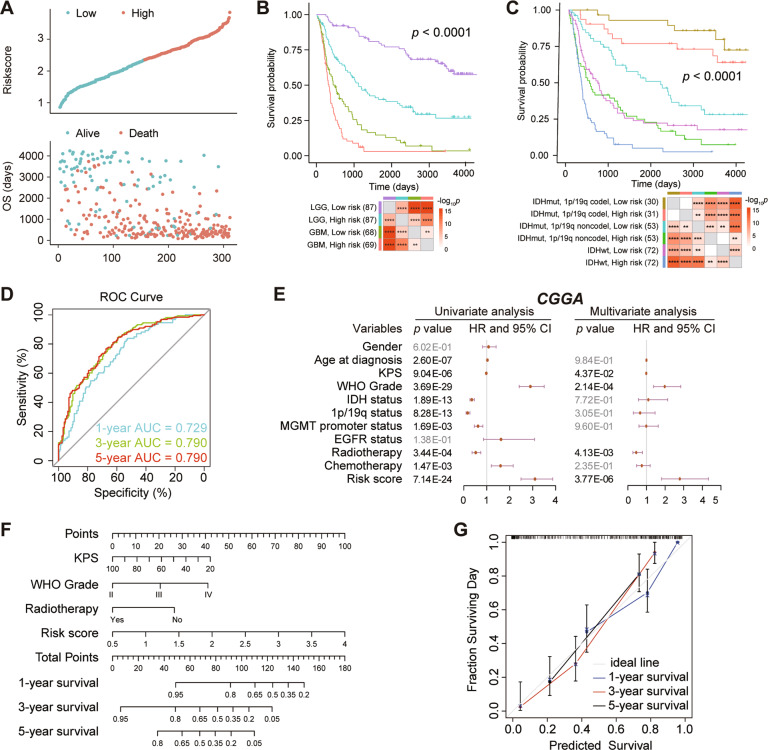
The prognostic value of the five-gene prognostic signature in CGGA dataset. **A** The risk score distribution (top) and survival status distribution (bottom) for glioma patients. **B** Kaplan–Meier survival curves for patients with glioma, classified into four groups based on grade and risk scores. **C** Kaplan–Meier survival curves for patients with glioma, classified by molecular pathological characteristic and risk scores. **D** ROC curves verified the prognostic performance of the risk score. **E** Univariate and multivariate Cox regression analyses of the association between clinic pathological factors and OS of patients. **F** Construction of a nomogram for survival prediction. **G** The calibration curve for the nomogram model. Three colored lines (blue, red and black) represent the performance of the nomogram. A closer fit to the diagonal gray line indicates a better estimation. ****p < 0.0001; ***p < 0.001; **p < 0.01.

Using the median risk score as a threshold, patients were distributed to high- and low-risk group in the CGGA training dataset. Patients with high-risk score had a higher mortality rate than those with low-risk score (Fig. 4A). LGG patients with the low-risk group showed the highest survival rates, whereas GBM patients with the high-risk group showed the lowest survival rates (*p* < 0.0001, Fig. 4B). When *IDH* mutation status, 1p/19q codeletion status and risk score were considered, patients within *IDH* mutation and 1p/19q codeletion in the low-risk group showed the best outcomes, whereas patients with *IDH*-wildtype in the high-risk group presented the worst prognosis (*p* < 0.0001, Fig. 4C). As shown in Fig. 4D, the area under the curve (AUC) reached 0.729 at 1 year, 0.790 at 3 years, and 0.790 at 5 years. In univariate Cox regression analysis, risk score was statistically related with OS in CGGA (*HR* = 3.10, *p* = 7.14E−24, Fig. 4E). After adjusting for other confounding factors, the risk score still remained an independent prognostic indicator for OS in patients with gliomas (CGGA: *HR* = 2.79, *p* = 3.77E−06, Fig. 4E). The similar results were validated in the TCGA (Supplementary Fig. S6A−E) and CGGA693 (Supplementary Fig. S7A−E) datasets. It indicates that risk score is an independent prognostic factor for OS.

Finally, the independent prognostic indicators for OS in multivariable Cox regression were selected and integrated to construct a prediction model. The risk score contributed the risk points from 0 to 100 in the CGGA dataset (Fig. 4F). The C-index for the prediction nomogram was 0.793 in the CGGA dataset. The calibration chart showed excellent agreement between the predictions and observations of the 1-year, 3-year, and 5-year probability of OS (Fig. 4G). It means that the signature is highly accurate.

### The risk signature is strongly associated with immune functions in glioma

**Fig. 5:**
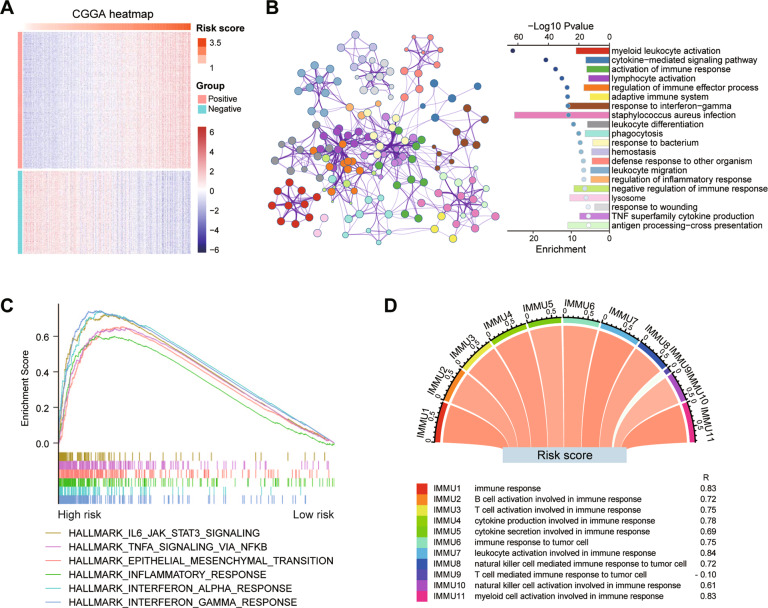
GO and GSEA annotation of genes associated with risk score in the CGGA dataset. **A** Heatmap of genes that are positively and negatively associated with risk score. **B** Functional enrichment of the positive related genes with the risk score by Metascape. **C** Enriched gene sets in HALLMARK collection by samples with high-risk score. **D** The correlation coefficient between risk score and different immune function scores. IMMU9 represents a negative correlation and other IMMNUs represent a positive correlation.

The Pearson correlation analysis was used to identify genes that were strongly positively (*R* > 0.7, *p* < 0.0001) or negatively (*R* < −0.6, *p* < 0.0001) correlated with the risk score in the CGGA and TCGA datasets. Totally, 685 and 1063 genes were separately identified in these two datasets (Fig. 5A and Supplementary Fig. S8A). By Metascape analysis, positively correlated genes were mainly enriched in the biological processes of ‘*myeloid leukocyte activation*’, ‘*cytokine-mediated signaling pathway*’, ‘*activation of immune response*’, and ‘*lymphocyte activation*’ (Fig. 5B and Supplementary Fig. S8B). While negatively correlated genes were involved in ‘*chemical synaptic transmission*’, ‘*synapse organization*’, and ‘*neuronal system*’ (Supplementary Fig. S9). In addition to interferon response and inflammatory response (Supplementary Table S3), the high-risk score group also enriched in ‘*IL6/JAK/STAT3 signaling*’, ‘*TNFα signaling via NFκB*’ and ‘*epithelial mesenchymal transition*’ in both CGGA and TCGA datasets by GSEA (Fig. 5C and Supplementary Fig. S8C). These results suggest that the risk score could be a good indicator of the immune response in glioma. Lastly, we performed a correlation analysis on the two datasets. Except for the term ‘*T cell mediated immune response to tumor cell*’, almost all immune functions are positively correlated with the risk score (Fig. 5D and Supplementary Fig. S8D).

### The high-risk score of signature predicate an enrichment of macrophages in glioma

**Fig. 6:**
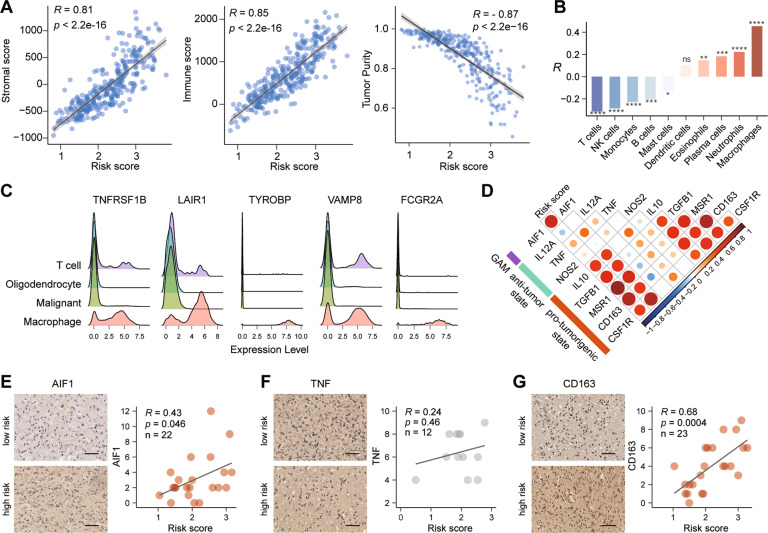
The relationship between risk score and tumor microenvironment in the CGGA dataset. **A** Scatter plots showed the relationship between stroma score, immune score, or tumor purity and risk score. **B** The correlation between immune infiltrating cells and the risk score. ****, *p* < 0.0001; ***, *p* < 0.001; **, *p* < 0.01; *, *p* < 0.05; ns, no significance. **C** Expression of five genes in single cells based on the GSE131928 database. **D** The correlation coefficient between risk score and glioma-associated microglia/macrophages markers. **E**−**G** The distribution of AIF1 (**E**), TNF (**F**) and CD163 (**G**) protein expression in the high- and low-risk groups by IHC staining. Scale bar, 50 μm.

To investigate the correlation between risk score and the TME, we first calculated the stromal and immune scores of each case in the CGGA and TCGA datasets. The results showed that both stromal and immune score were positively correlated with the risk score (Fig. 6A and Supplementary Fig. S10A). The tumor purity was negatively related with the risk score (Fig. 6A and Supplementary Fig. S10A). These results suggested that the proportion of infiltrating immune cells in gliomas increased along with the risk score increasing. Then, eight kinds of tumor-infiltrating immune cells (TICs) were significantly correlated with the risk score, and macrophages showed the strongest correlation (Fig. 6B, Supplementary Fig. S10B and Supplementary Table S4). These findings led us to speculate the relationship between our gene signature and glioma infiltrated macrophages, which promoted immunosuppression. An open single-cell sequencing dataset of glioblastomas demonstrated that these genes were highly expressed in macrophages, compared to other cells (Fig. 6C). Furthermore, general microglia/macrophage marker (*AIF1*) was significant positively correlated with risk score. In details, specific markers were selected to represent the polarization status of GAMs in glioma [32]. The correlation between risk score and anti-tumor state markers is inconsistent, but the risk score is positively correlated with pro-tumorigenic state markers (Fig. 6D and Supplementary Fig. S10C). Except TNF (*R* = 0.24, *p* = 0.46, *n* = 12, Fig. 6F), risk scores were significantly positively correlated with AIF1 (*R* = 0.43, *p* = 0.046, *n* = 22, Fig. 6E) and CD163 (*R* = 0.68, *p* = 0.0004, *n* = 23, Fig. 6G) by IHC assay. Taken together, these results indicated that samples with high-risk score showed an enrichment of GAMs in glioma, especially GAMs in pro-tumorigenic phenotype.

### The risk signature is associated with immune checkpoint and ICB response

**Fig. 7:**
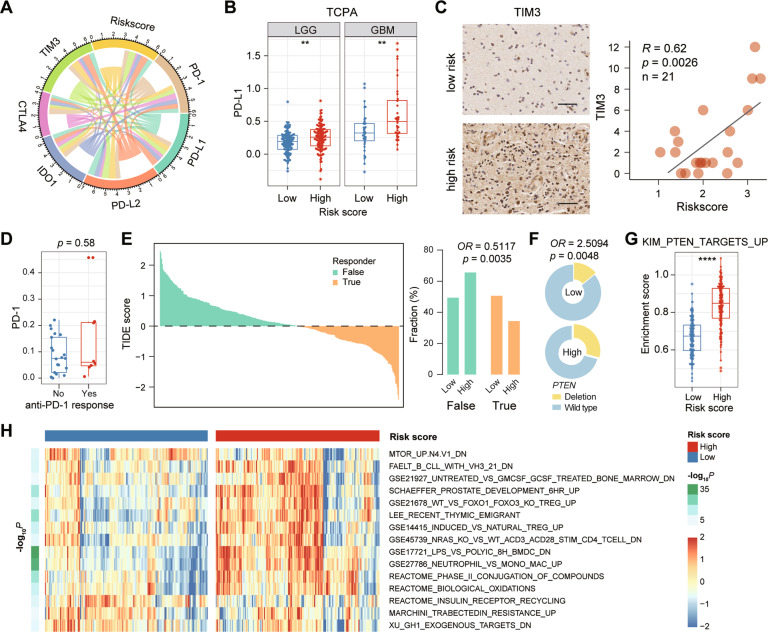
The relationship between risk score and tumor immune response. **A** The correlation coefficient between risk score and immune checkpoints. **B** The distribution of PD-L1 protein level in the high- and low-risk groups based on the TCPA datasets. **, *p* < 0.01. **C** The distribution of TIM3 protein level in the high- and low-risk groups by IHC staining. Scale bar, 50 μm. **D** The distribution of PD-1 expression in GBM patients before treatment with PD-1 inhibitors. **, *p* < 0.01. **E** The TIDE score and response results to immunotherapy of patients with glioma. **F** Fraction of *PTEN* loss in the low-risk group versus high-risk group. **G** GSEA enrichment score of gene-set KIM_PTEN_TARGETS_UP for the low-risk group versus high-risk group. ****, *p* < 0.0001. **H** Heatmap showing the gene sets differentially enriched in the low-risk group versus high-risk group.

Although ICB combination therapy has been shown to be effective in preclinical models of glioma, the efficacy in patient needs to be further verified [33]. Here, we first explored the relationship between the risk score of signature and the well-studied checkpoints. The results show that *PD-L1*, *PD-L2* and *TIM3* were positively correlated with the risk score (Fig. 7A). In addition, we also found that PD-L1 protein levels were higher in LGG and GBM patients with high-risk score from the TCPA database (Fig. 7B), and the expression of TIM3 protein was positively correlated with risk scores verified by the IHC assay (*R* = 0.62, *p* = 0.0026, *n* = 21, Fig. 7C). These results present a partial relationship between the risk signature and checkpoint expression. In a previous dataset [34], there was no difference in the distribution of *PD-1* expression between anti-PD-1 responders and non-responders before treatment with PD-1 inhibitors in GBM patients (Wilcoxon *p* = 0.58, Fig. 7D). Here, we investigate the relationship of patient’s response to ICB and risk score in the CGGA dataset by applying the Tumor Immune Dysfunction and Exclusion (TIDE) [23]. As results, patients in the low-risk score group (50.6%, 82/162) were more likely to respond to ICB therapy than those in the high-risk score group (34.4%, 56/163) (Fisher *p* = 0.0035, Fig. 7E). In addition, we found more frequent *PTEN* loss (Fisher *p* = 0.0048, Fig. 7F) and higher PI3K-Akt pathway activity (*p* < 0.0001, Fig. 7G) in high-risk patients, consistent with these molecular events being identified as not benefiting from PD1 inhibitors [34]. Zhao et al. [34] have identified a series of gene sets differentially enriched in responders versus non-responders before anti-PD-1 immunotherapy. By ssGSEA, we found that these gene sets also showed significant differences between the low-risk and high-risk groups (Fig. 7H). In summary, these results indicated that glioma with the high-risk group might be less responsive to ICB therapy, especially anti-PD-1 immunotherapy.

## Discussion

In this study, we found the unique gene signature in each subtype of glioma, which is based on a large-scale gene expression profile and integrated analysis. Turquoise and yellow modules were significantly positive correlated with GBM, IDH-wildtype, which is the most malignant subtype in glioma (Fig. 1A). The biological function and upstream TFs of these modules are significantly distinct. Genes in turquoise module participated in immune process and mainly regulated by *NFKB1*, *RELA*, *SP1*, *STAT1*, and *STAT3* (Fig. 1D, E). Activation of NF-κB induced transcription of cytokines, cell cycle, apoptosis, and angiogenesis factors, which are drivers in tumor-promoting environment [35]. STAT3 is constitutively activated both in tumor cells and TICs. Activated STAT3 not only triggers glioma progression through affecting cell proliferation, apoptosis, invasion, and angiogenesis, but also functions as an inducer of immune evasion within GBM microenvironment [36]. Genes in yellow module are involved in cell cycle and cell division and regulated by E2Fs and TP53 (Fig. 1D, E). The CDKs/RB/E2Fs signaling pathway is a major transcriptional machinery of cell-cycle-dependent gene expression [37]. Owing to inactivation of RB, amplification of CDKs or deletion of CDK inhibitors, E2Fs activity is high in virtually all cancers [37]. Inactivation of ARF/MDM2/TP53 signaling pathway occurred in 87% GBM patients, including deletion or mutation of *ARF*, amplifications of *MDM2* or *MDM4*, deletion or mutation of *TP53* [38]. Our results verified that immune response and cell-cycle dysfunction are two core deregulated processes in the pathogenesis of glioma and regulated by distinct upstream pathways.

By comprehensive bioinformatic analysis, a five-immune gene signature, including *TNFRSF1B, LAIR1, TYROBP, VAMP8,* and *FCGR2A*, was constructed (Fig. 1F–H). TNFRSF1B, a receptor of TNF and lymphotoxia-α, can directly promote tumor cell proliferation and activate immunosuppressive cells [27]. LAIR1 is an immune inhibitory receptor, plays a negative role in solid tumor growth, and the activation of LAIR1 on immune cells may lead to suppression of anti-tumor immune responses [28]. TYROBP is a transmembrane signaling polypeptide and triggers receptors on the immune cells’ surface [39]. TYROBP expression was significantly higher in the LGG tissues compared to the normal tissues and associated with worse prognosis and poor clinic pathological parameters in glioma [29]. VAMP8 is a SNARE protein, which function as an oncogene by promoting cell proliferation and therapeutic resistance in glioma [30]. FCGR2A is a cell surface receptor on phagocytic cells, involved in the process of phagocytosis and clearance of immune complexes [40]. Silencing *FCGR2A* expression suppressed glioma proliferation, migration and invasion [31]. Collectively, the function of these genes in tumors, especially VAMP8 and FCGR2A in gliomas, has been fully demonstrated.

The high-risk score showed an enrichment of well-known malignant features, which indicated that the high-risk score may predict poor outcome of glioma patients (Fig. 2). As expected, the risk score of our signature was significantly related to OS of glioma patients in three independent datasets including 1619 cases (Fig. 4, and Supplementary Figs. S6, S7). Our five-immune gene signature showed a favorable efficiency in stratified subgroups, which are divided by WHO grade, *IDH* and 1p/19q status. In addition, after adjusting for other confounding factors, the risk score of signature is an independent predictor of OS in patients with glioma. These indicated that the risk score showed good predictions for the outcome of different datasets and multiple subgroups.

The GO analysis showed that positive genes related with risk score were enriched in immune-related responses (Fig. 5B), which play decisive roles in different stages of tumor development and affect the response to therapy [41]. The GSEA analysis has showed that NF-κB signaling, Jak-Stat3 signaling, and epithelial-mesenchymal transitions (EMT) are correlated with the high-risk score group (Fig. 5C), which are critical mediators of tumor invasion and immune evasion. The risk score of our immune signature is positively correlated with almost all immune functions (Fig. 5D). Taken together, these results confirmed the important role of our signature in immune response and suggested that genes in signature may affect the glioma microenvironment.

Then, a higher complexity of immune cells was showed in high-risk patients, and the risk score was significantly positively correlated with the abundance of macrophages (Fig. 6B). In addition, we found that genes in our signature are highly expressed in macrophages by an open single-cell sequencing dataset of GBM (Fig. 6C), which may partly explain the correlation between our signature and macrophage infiltration in glioma. By correlation analysis and IHC assay (Fig. 6D–G), we further demonstrated that patients with a high-risk score showed an enrichment of macrophage in a tumor-supportive state, but not an anti-tumor state.

Immune checkpoint inhibitors therapy, especially those targeting PD-1/PD-L1 and CTLA-4, have showed an efficacy in several tumor types. However, less than 10% of GBM patients show long-term response. We wondered that whether the score of our immune signature can predict the glioma patient response to ICB therapy. First, the risk score of signature showed a positive correlation with immune checkpoints (Fig. 7A), especially *PD-L1* [42], *PD-L2* [43], *TIM3* [44]. By TCPA and IHC assay, we verified that the high-risk group showed a higher expression of PD-L1 and TIM3 protein (Fig. 7B, C). These results indicated that high-risk score might predict an immune suppressive environment in tumor. In current research community in glioma, however, there are no markers that can be used to predict responses to immunotherapy of glioma patients. In this study, we used TIDE algorithm to predict the possibility of patient’s response to ICB therapy. As result, the low-risk group is more likely to respond to ICB therapy (Fig. 7E). With high frequency of *PTEN* deletions and PI3K-AKT pathway activity (Fig. 7F, G), the high-risk group may not respond to PD-1 inhibitors. Consistent with previous study, glioblastoma revealed a significant enrichment of *PTEN* mutations associated with immunosuppressive expression signatures in PD-1 inhibitors non-responders [34]. These results indicated that our signature can be used to predict the response of glioma patients after receiving ICB treatment, especially anti-PD-1 immunotherapy, thereby further broadening the scope of application of this signature.

In summary, we constructed a gene co-expression network of glioma, and analyzed the functional features and upstream TFs of these modules. An immune gene signature was constructed and showed a favorable efficiency in predicting prognosis of patients with glioma. High-risk score of this signature predicted an enrichment of macrophages and less response to ICB therapy in glioma. Notably, our five-immune gene signature may help to develop personalized treatment plans or estimate whether patients can benefit from ICB treatment, especially anti-PD-1 immunotherapy.

## Supplementary information

SUPPLEMENTAL MATERIAL

aj-checklist

## Data Availability

All data generated and analyzed during this study are included in this published article and its additional files.

## References

[1] Ostrom QT, Cioffi G, Gittleman H, Patil N, Waite K, Kruchko C (2019). CBTRUS statistical report: primary brain and other central nervous system tumors diagnosed in the United States in 2012−6. Neuro Oncol.

[2] Jiang T, Nam DH, Ram Z, Poon WS, Wang J, Boldbaatar D (2021). Clinical practice guidelines for the management of adult diffuse gliomas. Cancer Lett.

[3] Louis DN, Perry A, Reifenberger G, von Deimling A, Figarella-Branger D, Cavenee WK (2016). The 2016 World Health Organization Classification of Tumors of the Central Nervous System: a summary. Acta Neuropathol.

[4] Brat DJ, Aldape K, Colman H, Holland EC, Louis DN, Jenkins RB (2018). cIMPACT-NOW update 3: recommended diagnostic criteria for “Diffuse astrocytic glioma, IDH-wildtype, with molecular features of glioblastoma, WHO grade IV”. Acta Neuropathol.

[5] Hu H, Mu Q, Bao Z, Chen Y, Liu Y, Chen J (2018). Mutational landscape of secondary glioblastoma guides MET-targeted trial in brain tumor. Cell.

[6] Quail DF, Joyce JA (2013). Microenvironmental regulation of tumor progression and metastasis. Nat Med.

[7] Quail DF, Joyce JA (2017). The microenvironmental landscape of brain tumors. Cancer Cell.

[8] Wei J, Chen P, Gupta P, Ott M, Zamler D, Kassab C (2020). Immune biology of glioma-associated macrophages and microglia: functional and therapeutic implications. Neuro Oncol.

[9] Mantovani A, Sozzani S, Locati M, Allavena P, Sica A (2002). Macrophage polarization: tumor-associated macrophages as a paradigm for polarized M2 mononuclear phagocytes. Trends Immunol.

[10] Komohara Y, Ohnishi K, Kuratsu J, Takeya M (2008). Possible involvement of the M2 anti-inflammatory macrophage phenotype in growth of human gliomas. J Pathol.

[11] Hambardzumyan D, Gutmann DH, Kettenmann H (2016). The role of microglia and macrophages in glioma maintenance and progression. Nat Neurosci.

[12] Zhao Z, Meng F, Wang W, Wang Z, Zhang C, Jiang T (2017). Comprehensive RNA-seq transcriptomic profiling in the malignant progression of gliomas. Sci Data.

[13] Zhao Z, Zhang KN, Wang Q, Li G, Zeng F, Zhang Y (2021). Chinese Glioma Genome Atlas (CGGA): a comprehensive resource with functional genomic data from Chinese glioma patients. Genomics Proteom Bioinform.

[14] Langfelder P, Horvath S (2008). WGCNA: an R package for weighted correlation network analysis. BMC Bioinform.

[15] Han H, Cho JW, Lee S, Yun A, Kim H, Bae D (2018). TRRUST v2: an expanded reference database of human and mouse transcriptional regulatory interactions. Nucleic Acids Res.

[16] Zhou Y, Zhou B, Pache L, Chang M, Khodabakhshi AH, Tanaseichuk O (2019). Metascape provides a biologist-oriented resource for the analysis of systems-level datasets. Nat Commun.

[17] Baysan M, Bozdag S, Cam M, Kotliarova S, Ahn S, Walling J (2012). G-cimp status prediction of glioblastoma samples using mRNA expression data. PLoS ONE.

[18] Noushmehr H, Weisenberger DJ, Diefes K, Phillips HS, Pujara K, Berman BP (2010). Identification of a CpG island methylator phenotype that defines a distinct subgroup of glioma. Cancer Cell.

[19] Stuart T, Butler A, Hoffman P, Hafemeister C, Papalexi E, Mauck WM (2019). Comprehensive integration of single-cell data. Cell.

[20] Valastyan S, Weinberg RA (2011). Tumor metastasis: molecular insights and evolving paradigms. Cell.

[21] Zhang C, Cheng W, Ren X, Wang Z, Liu X, Li G (2017). Tumor purity as an underlying key factor in glioma. Clin Cancer Res.

[22] Newman AM, Liu CL, Green MR, Gentles AJ, Feng W, Xu Y (2015). Robust enumeration of cell subsets from tissue expression profiles. Nat Methods.

[23] Jiang P, Gu S, Pan D, Fu J, Sahu A, Hu X (2018). Signatures of T cell dysfunction and exclusion predict cancer immunotherapy response. Nat Med.

[24] Zeng F, Wang K, Liu X, Zhao Z (2020). Comprehensive profiling identifies a novel signature with robust predictive value and reveals the potential drug resistance mechanism in glioma. Cell Commun Signal.

[25] Ma W, Zhang K, Bao Z, Jiang T, Zhang Y (2021). SAMD9 is relating with M2 macrophage and remarkable malignancy characters in low-grade glioma. Front Immunol.

[26] Bader GD, Hogue CW (2003). An automated method for finding molecular complexes in large protein interaction networks. BMC Bioinform.

[27] Sheng Y, Li F, Qin Z (2018). TNF receptor 2 makes tumor necrosis factor a friend of tumors. Front Immunol.

[28] Guo N, Zhang K, Gao X, Lv M, Luan J, Hu Z (2020). Role and mechanism of LAIR-1 in the development of autoimmune diseases, tumors, and malaria: a review. Curr Res Transl Med.

[29] Lu J, Peng Y, Huang R, Feng Z, Fan Y, Wang H (2021). Elevated TYROBP expression predicts poor prognosis and high tumor immune infiltration in patients with low-grade glioma. BMC Cancer.

[30] Chen Y, Meng D, Wang H, Sun R, Wang D, Wang S (2015). VAMP8 facilitates cellular proliferation and temozolomide resistance in human glioma cells. Neuro Oncol.

[31] Zhang Z, Chen J, Huo X, Zong G, Huang K, Cheng M (2021). Identification of a mesenchymal-related signature associated with clinical prognosis in glioma. Aging.

[32] Gabrusiewicz K, Rodriguez B, Wei J, Hashimoto Y, Healy LM, Maiti SN (2016). Glioblastoma-infiltrated innate immune cells resemble M0 macrophage phenotype. JCI Insight.

[33] Wang X, Lu J, Guo G, Yu J (2021). Immunotherapy for recurrent glioblastoma: practical insights and challenging prospects. Cell Death Dis.

[34] Zhao J, Chen AX, Gartrell RD, Silverman AM, Aparicio L, Chu T (2019). Immune and genomic correlates of response to anti-PD-1 immunotherapy in glioblastoma. Nat Med.

[35] Concetti J, Wilson CL (2018). NFKB1 and cancer: friend or foe?. Cells.

[36] Chang N, Ahn SH, Kong DS, Lee HW, Nam DH (2017). The role of STAT3 in glioblastoma progression through dual influences on tumor cells and the immune microenvironment. Mol Cell Endocrinol.

[37] Kent LN, Leone G (2019). The broken cycle: E2F dysfunction in cancer. Nat Rev Cancer.

[38] Cancer Genome Atlas Research Network. Comprehensive genomic characterization defines human glioblastoma genes and core pathways. Nature. 2008;455:1061−8. 10.1038/nature07385 PMC2671642 18772890

[39] Painter MM, Atagi Y, Liu CC, Rademakers R, Xu H, Fryer JD (2015). TREM2 in CNS homeostasis and neurodegenerative disease. Mol Neurodegener.

[40] Arman M, Krauel K (2015). Human platelet IgG Fc receptor FcγRIIA in immunity and thrombosis. J Thromb Haemost.

[41] Grivennikov SI, Greten FR, Karin M (2010). Immunity. Inflamm, Cancer Cell.

[42] DiDomenico J, Lamano JB, Oyon D, Li Y, Veliceasa D, Kaur G (2018). The immune checkpoint protein PD-L1 induces and maintains regulatory T cells in glioblastoma. Oncoimmunology.

[43] Wang ZL, Li GZ, Wang QW, Bao ZS, Wang Z, Zhang CB (2019). PD-L2 expression is correlated with the molecular and clinical features of glioma, and acts as an unfavorable prognostic factor. Oncoimmunology.

[44] Li G, Wang Z, Zhang C, Liu X, Cai J, Wang Z (2017). Molecular and clinical characterization of TIM-3 in glioma through 1,024 samples. Oncoimmunology.

