# Supplementary material for: Comprehensive transcriptomic characterization reveals core genes and module associated with immunological changes via 1619 samples of brain glioma
Source: Cell Death Dis. 2021 Dec 8;12(12):1140. doi: 10.1038/s41419-021-04427-8 (PMC8654825; doi:10.1038/s41419-021-04427-8)
Supplement: Supplementary file 1 — SUPPLEMENTAL MATERIAL [file 41419_2021_4427_MOESM1_ESM.docx]

**Supplemental material**

**Comprehensive transcriptomic characterization reveals core genes and module associated with immunological changes via 1,619 samples of brain glioma**

**
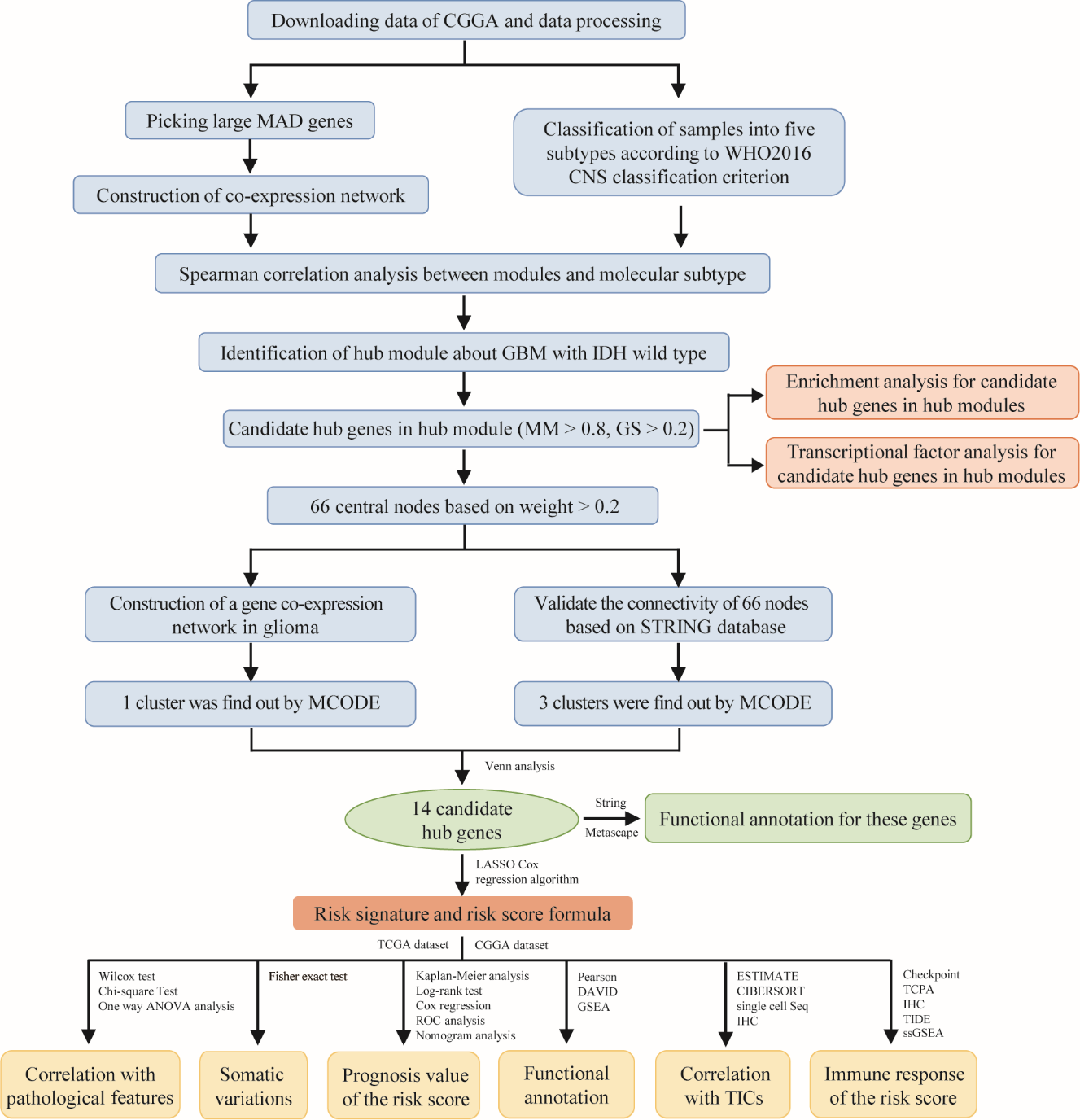
**

**Supplement figure 1.** Flow chart of the study.

**
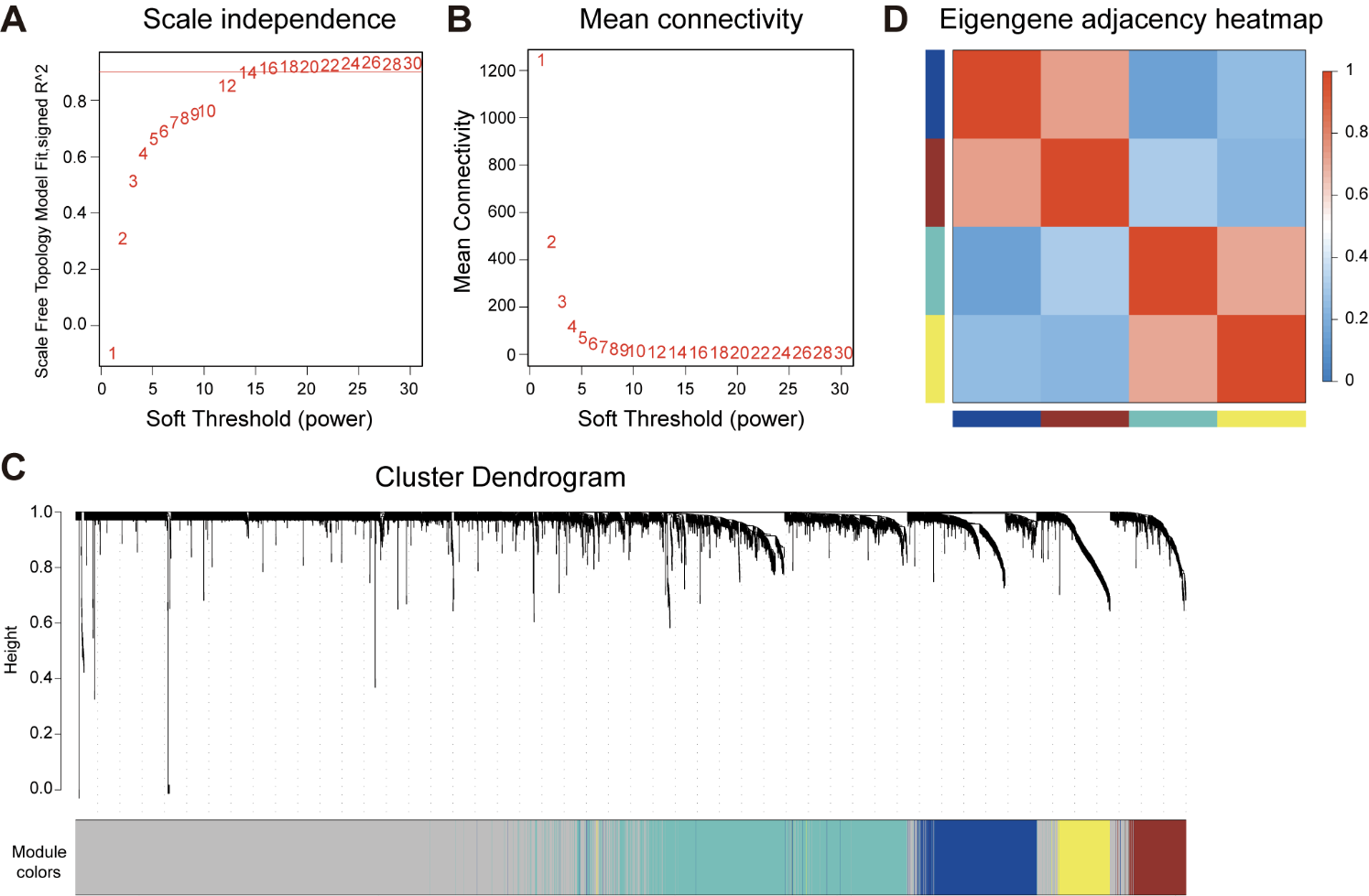
**

**Supplement figure 2.** Gene co-expression network construction in glioma. (A) Analyze the scale-free fit index of the 1-30 soft threshold power (β). (B) Analyze the average connectivity of 1-30 soft threshold power. (C) Hierarchical clustering tree of 5000 genes in various modules. Each colored row represents a color-coded module which contains a group of highly connected genes. (D) The eigengene adjacency heatmap of 4 co-expression modules.

**
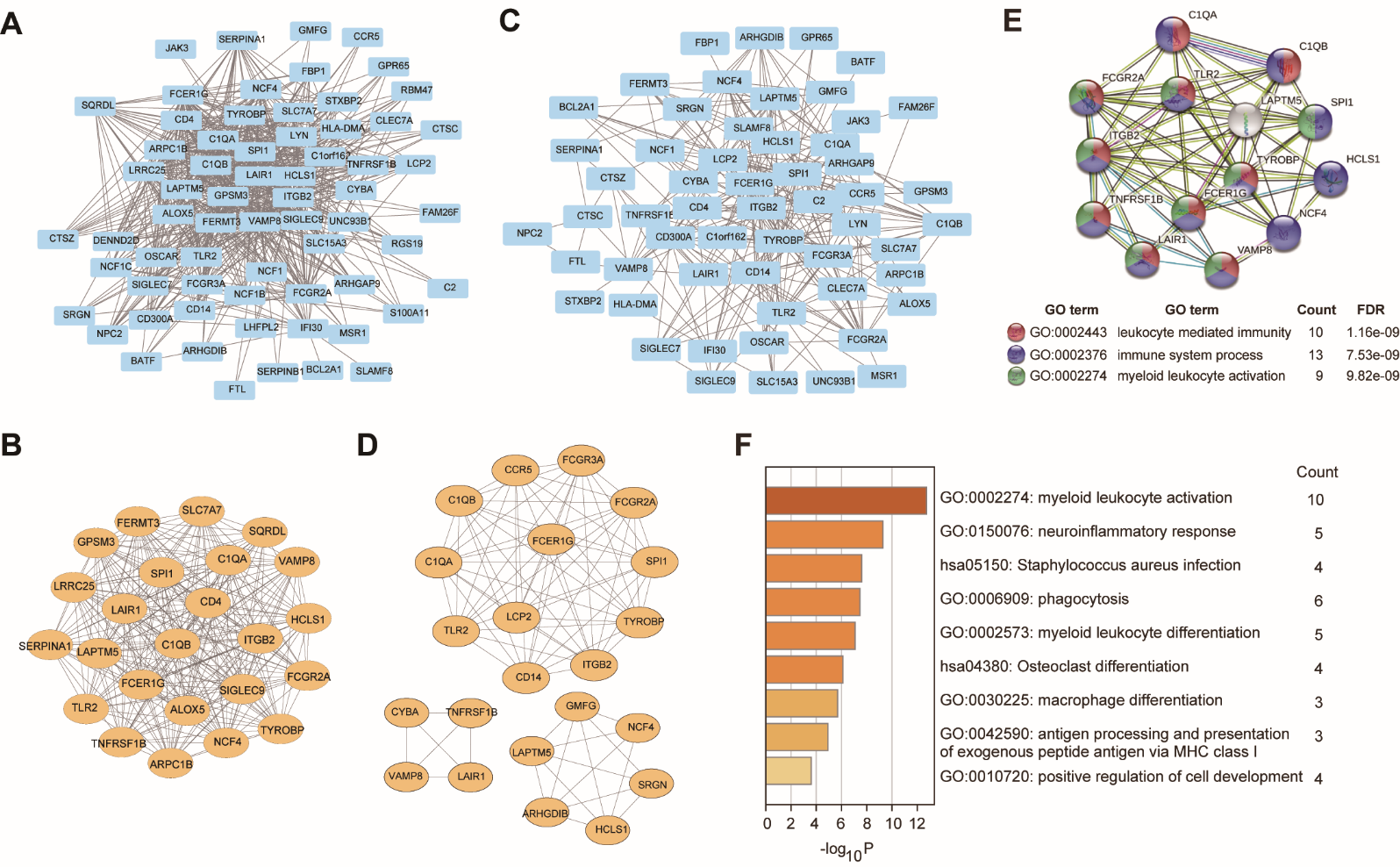
**

**Supplement figure 3.** Identification of 14 hub genes in turquoise module. (A) PPI network of highly connected genes in turquoise module. In this network, we only display connections whose topological overlaps above the threshold 0.2. (B) The core cluster genes in turquoise module obtained by MCODE analysis. (C) The PPI network of 66 genes in STRING. (D) The core cluster genes in STRING obtained by MCODE analysis. (E-F) Functional annotation of the 14 hub genes by STRING (E) and Metascape (F) analysis.


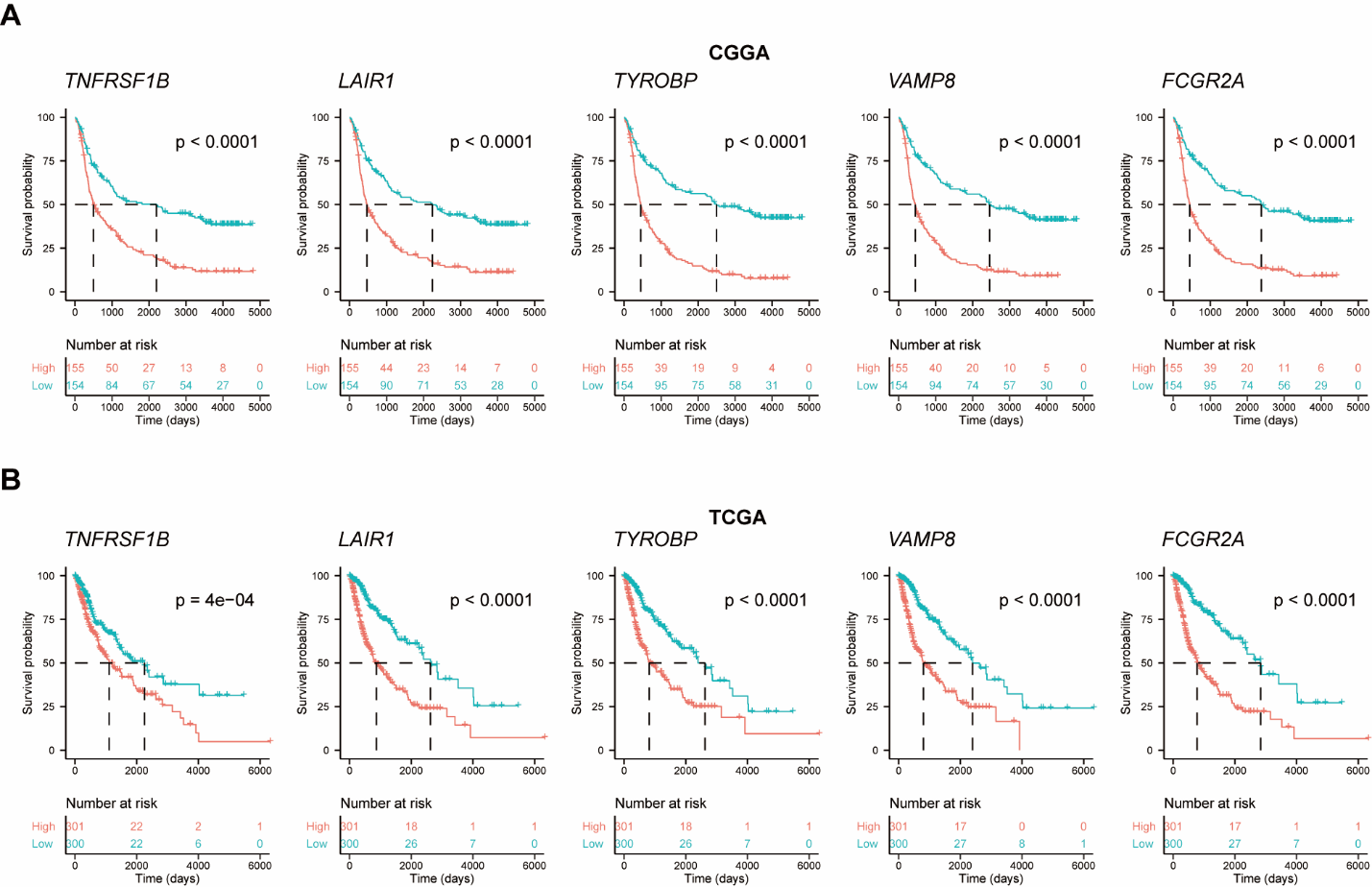


**Supplement figure 4.** The prognostic performance of *TNFRSF1B, LAIR1, TYROBP, VAMP8* and *FCGR2A* in CGGA (A) and TCGA (B) datasets.

**
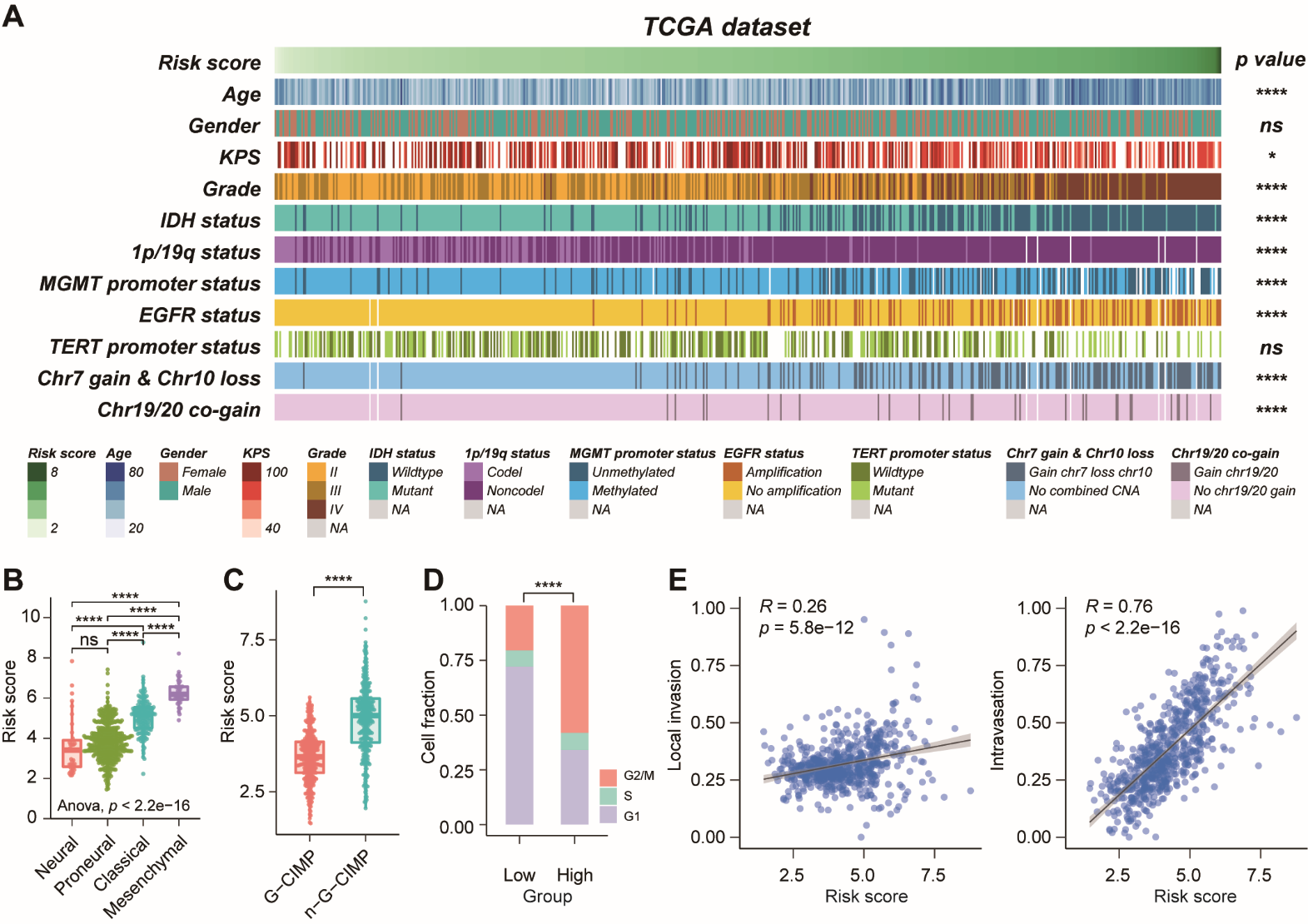
**

**Supplement figure 5.** Relationship between the signature risk score and the pathological characteristics in TCGA dataset. (A) The distribution of clinical and pathological characteristics arranged by the increasing risk score. (B) Distribution of risk score in patients stratified by TCGA subtype. (C) Distribution of risk score in patients stratified by G-CIMP subtype. (D) The relationship between risk score and cell cycle proportion in TCGA dataset. (E) The correlation between risk score and invasion index were analyzed by Pearson correlation analysis. ****, *p* < 0.0001; *, *p* < 0.05; ns, no significance.

**
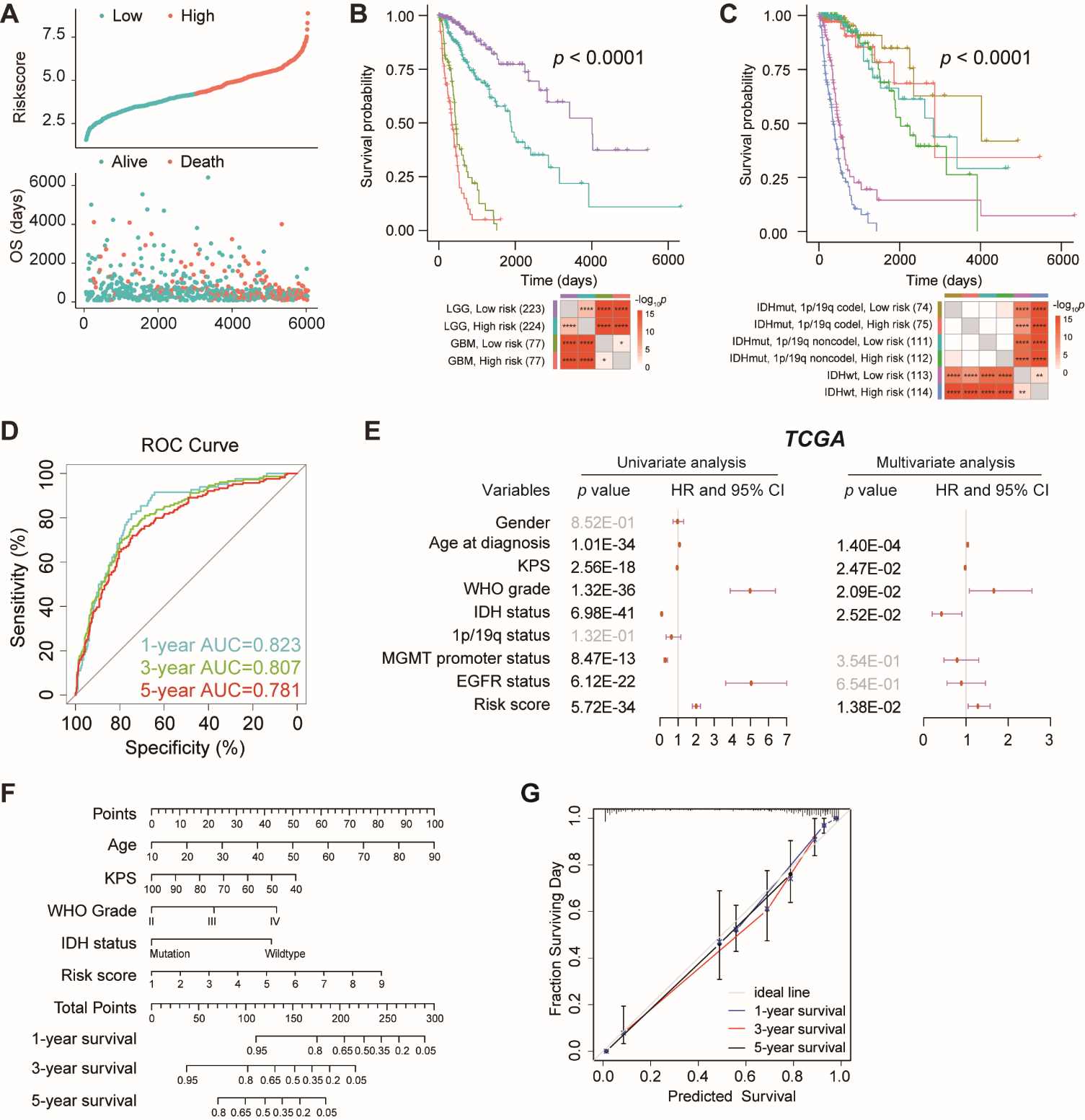
**

**Supplement figure 6.** Validation the prognostic performance of the 5-gene prognostic signature in TCGA dataset. (A) The risk score distribution (top) and survival status distribution (bottom) for glioma patients. (B) Kaplan–Meier survival curves for patients with glioma, classiﬁed into four groups based on grade and risk scores. (C) Kaplan–Meier survival curves for patients with glioma, classiﬁed by molecular pathological characteristic and risk scores. (D) ROC curves verified the prognostic performance of the risk score. (E) Univariate and multivariate Cox regression analyses of the association between clinic pathological factors and OS of patients. (F) Construction of a nomogram for survival prediction. (G) The calibration curve for the nomogram model. Three colored lines (blue, red and black) represent the performance of the nomogram. A closer fit to the diagonal gray line indicates a better estimation. ****, *p* < 0.0001; ***, *p* < 0.001; **, *p* < 0.01; *, *p* < 0.05.

**
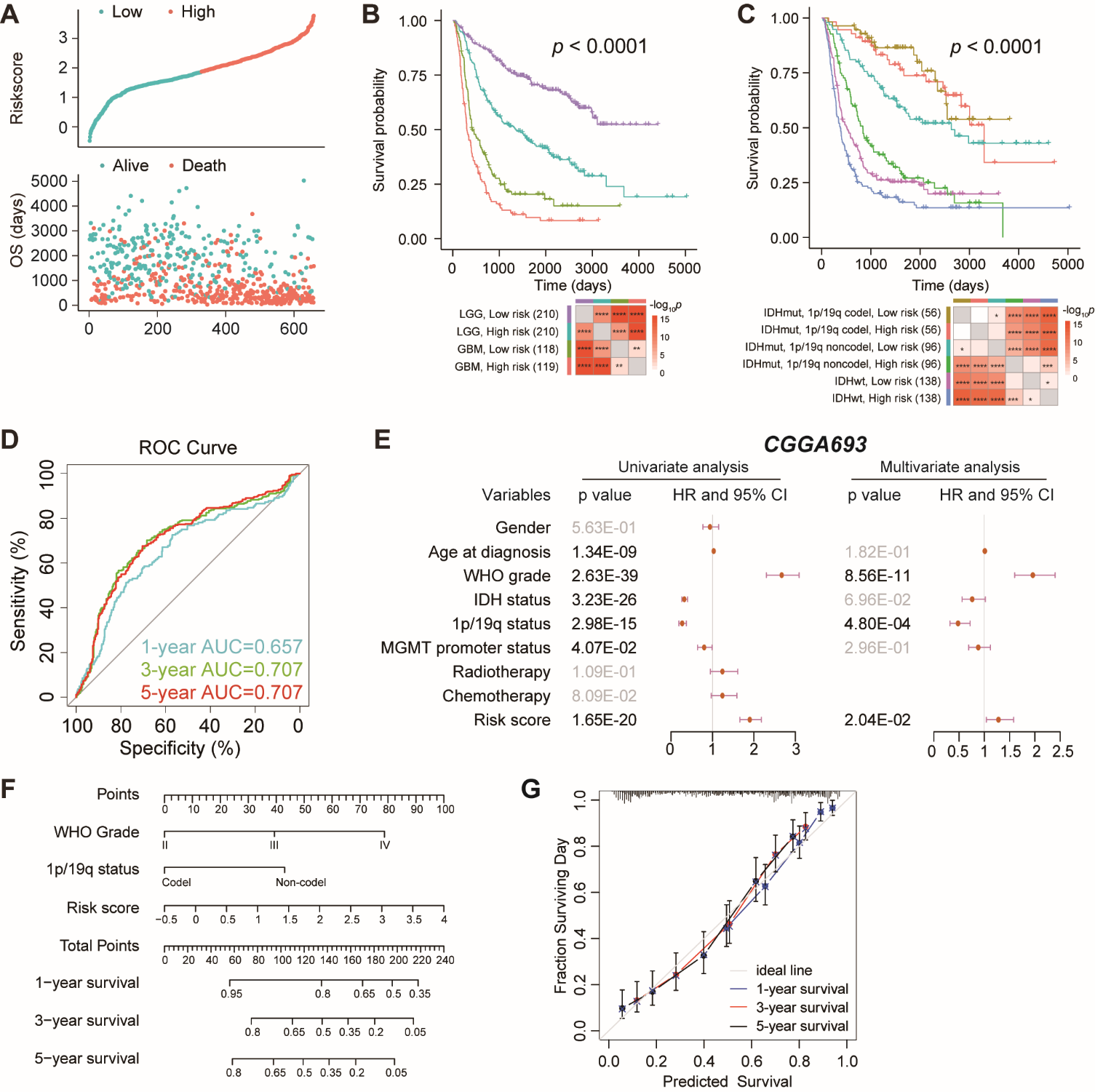
**

**Supplement figure 7.** Validation the prognostic performance of the 5-gene prognostic signature in CGGA693 dataset. (A) The risk score distribution (top) and survival status distribution (bottom) for glioma patients. (B) Kaplan–Meier survival curves for patients with glioma, classiﬁed into four groups based on grade and risk scores. (C) Kaplan–Meier survival curves for patients with glioma, classiﬁed by molecular pathological characteristic and risk scores. (D) ROC curves verified the prognostic performance of the risk score. (E) Univariate and multivariate Cox regression analyses of the association between clinic pathological factors and OS of patients. (F) Construction of a nomogram for survival prediction. (G) The calibration curve for the nomogram model. Three colored lines (blue, red and black) represent the performance of the nomogram. A closer fit to the diagonal gray line indicates a better estimation. ****, *p* < 0.0001; ***, *p* < 0.001; **, *p* < 0.01; *, *p* < 0.05.


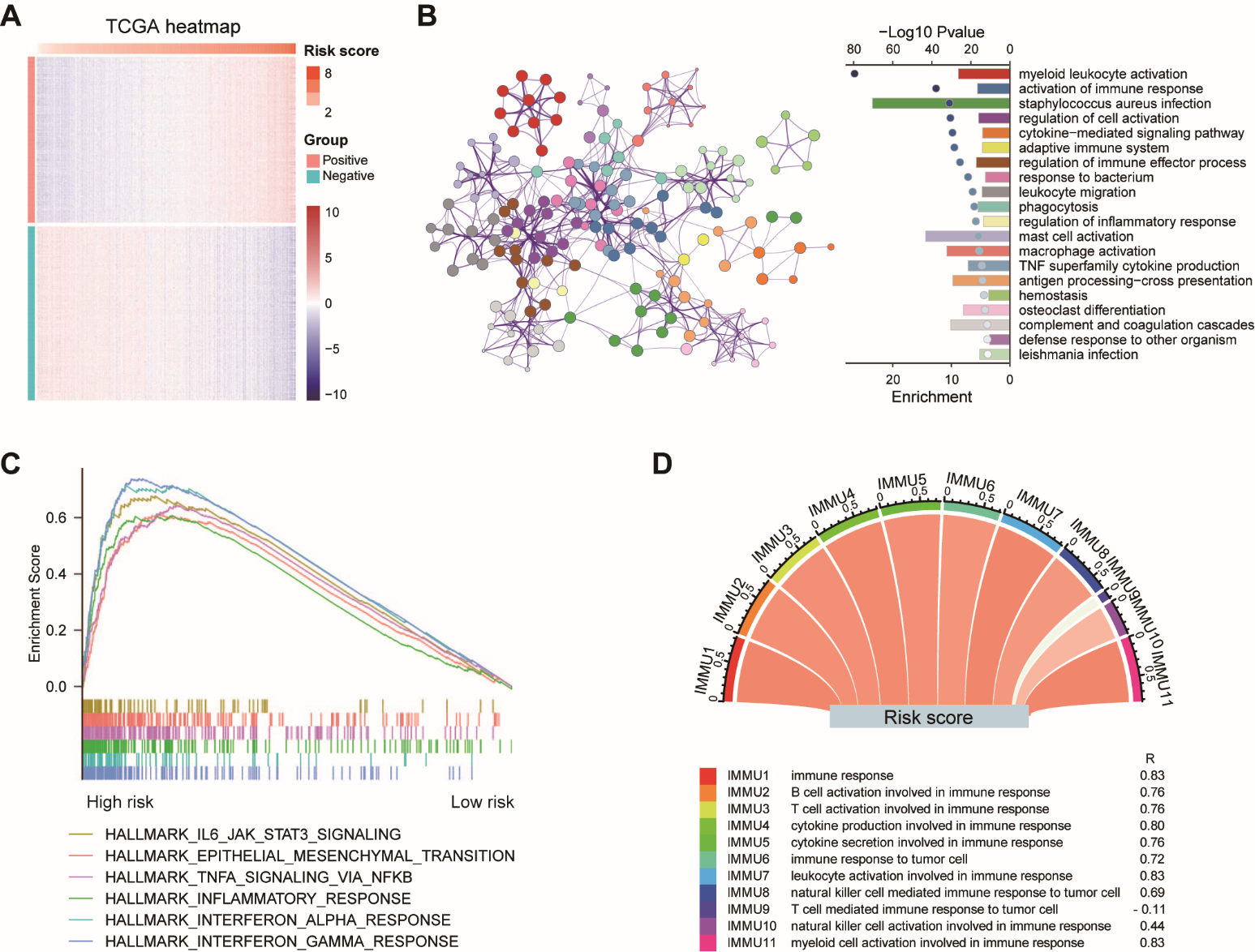


**Supplement figure 8.** GO and GSEA annotation of genes associated with risk score in TCGA dataset. (A) Heatmap of genes that are positively and negatively associated with risk score. (B) Functional enrichment of the positive related genes with the risk score by Metascape. (C) Enriched gene sets in HALLMARK collection by samples with high risk score. (D) The correlation coefﬁcient between risk score and different immune function scores. IMMU9 represent a negative correlation and other IMMUs represent a positive correlation.


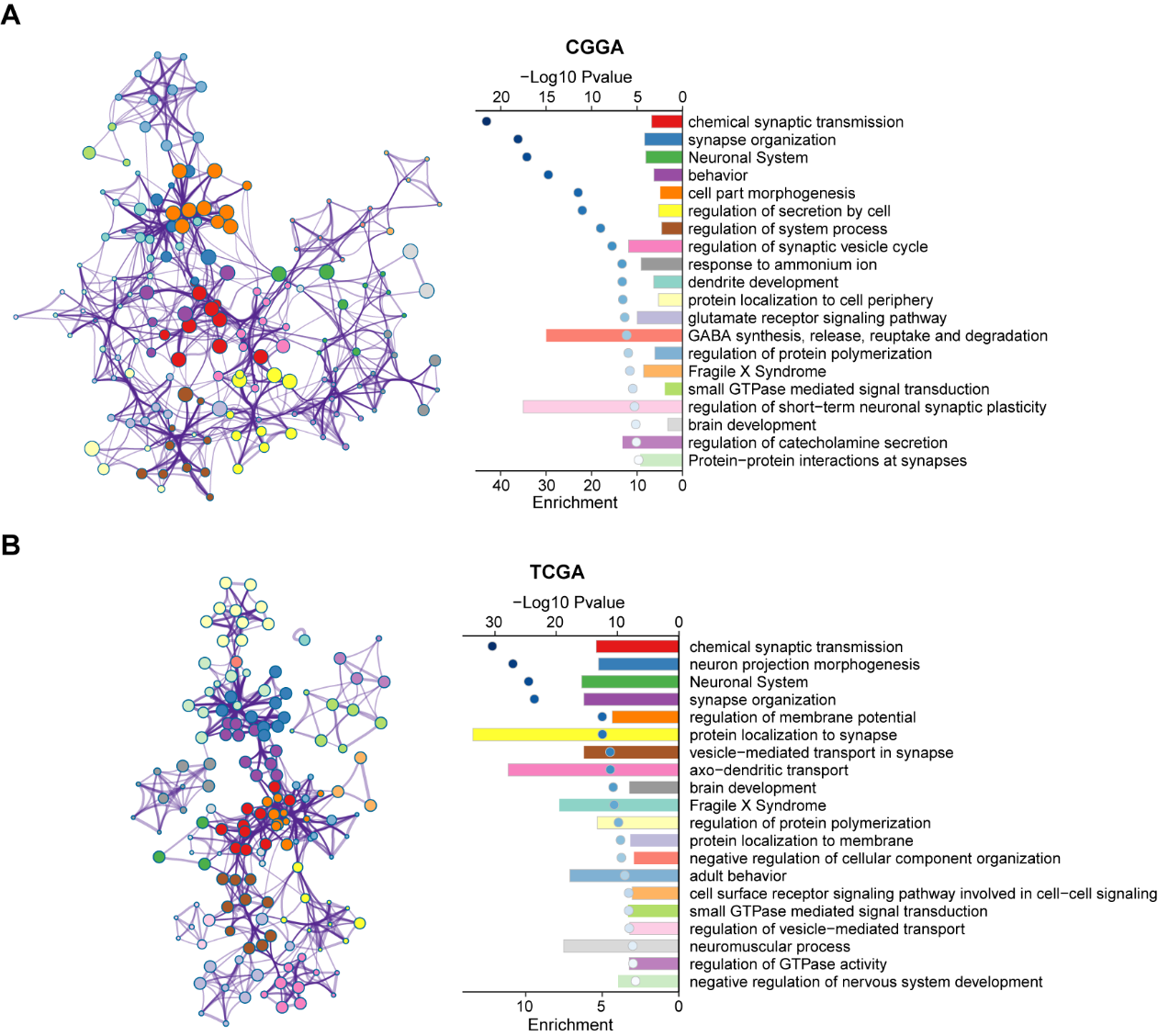


**Supplement figure 9.** Functional enrichment of negative related genes with the risk score by Metascape in CGGA (A) and TCGA (B) datasets.


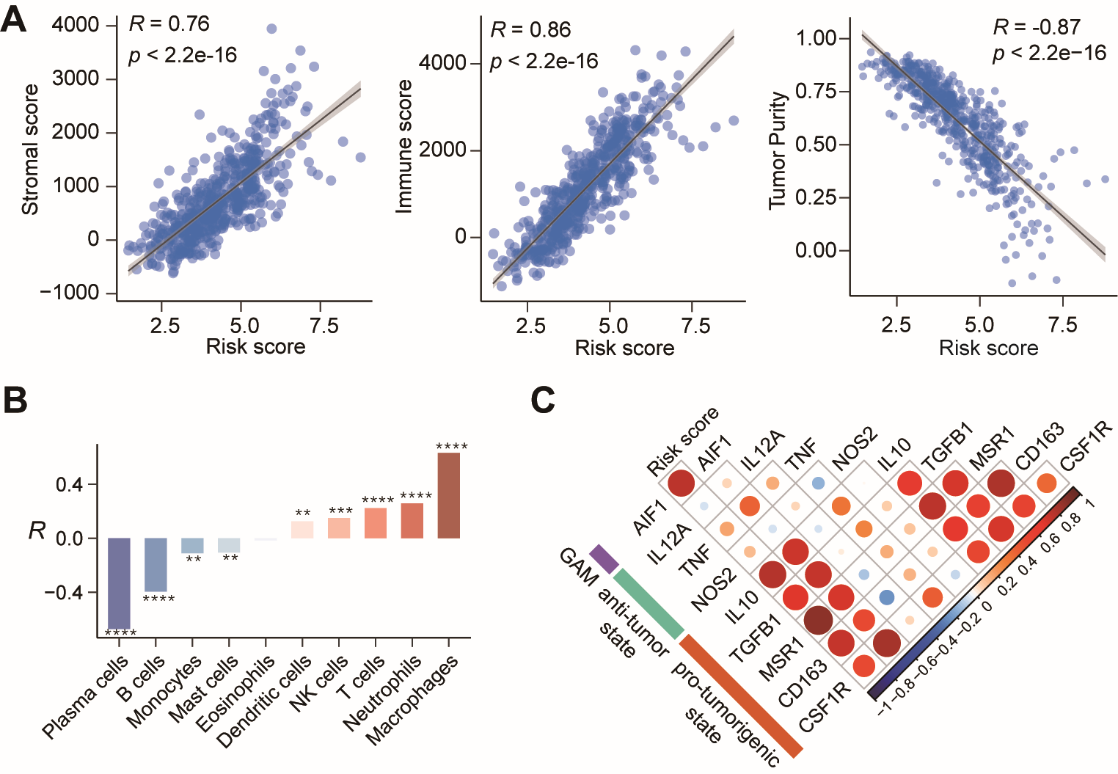


**Supplement figure 10.** The relationship between risk score and tumor microenvironment in in TCGA dataset. (A) Scatter plots showed the relationship between stroma score, immune score, or tumor purity and risk score. (B) The correlation between immune infiltrating cells and the risk score. (C) The correlation coefﬁcient between risk score and glioma-associated microglia/macrophages markers.

**Supplement table 1** Key transcriptional factor for turquoise module.

| Key TF | Description | Targets | P value | FDR |
| --- | --- | --- | --- | --- |
| NFKB1 | nuclear factor of kappa light polypeptide gene enhancer in B-cells 1 | 27 | 4.11E-14 | 2.75E-12 |
| RELA | v-rel reticuloendotheliosis viral oncogene homolog A (avian) | 26 | 2.59E-13 | 8.67E-12 |
| SP1 | Sp1 transcription factor | 28 | 2.46E-10 | 5.49E-09 |
| STAT1 | signal transducer and activator of transcription 1, 91kDa | 12 | 2.81E-09 | 4.70E-08 |
| STAT3 | signal transducer and activator of transcription 3 (acute-phase response factor) | 12 | 1.03E-06 | 1.38E-05 |
| USF1 | upstream transcription factor 1 | 8 | 4.15E-06 | 4.64E-05 |
| CEBPA | CCAAT/enhancer binding protein (C/EBP), alpha | 7 | 8.04E-06 | 7.69E-05 |
| HOXD3 | homeobox D3 | 3 | 2.90E-05 | 2.43E-04 |
| SP3 | Sp3 transcription factor | 9 | 3.78E-05 | 2.82E-04 |
| FOSL2 | FOS-like antigen 2 | 4 | 5.08E-05 | 3.41E-04 |
| JUN | jun proto-oncogene | 10 | 6.14E-05 | 3.74E-04 |
| JUND | jun D proto-oncogene | 5 | 1.18E-04 | 6.61E-04 |
| KLF8 | Kruppel-like factor 8 | 3 | 1.57E-04 | 8.10E-04 |
| HIF1A | hypoxia inducible factor 1, alpha subunit (basic helix-loop-helix transcription factor) | 7 | 1.95E-04 | 9.32E-04 |
| SPI1 | spleen focus forming virus (SFFV) proviral integration oncogene spi1 | 6 | 2.65E-04 | 1.18E-03 |
| PARP1 | poly (ADP-ribose) polymerase 1 | 4 | 4.20E-04 | 1.66E-03 |
| SRF | serum response factor (c-fos serum response element-binding transcription factor) | 4 | 4.20E-04 | 1.66E-03 |
| USF2 | upstream transcription factor 2, c-fos interacting | 5 | 5.60E-04 | 2.01E-03 |
| ERG | v-ets erythroblastosis virus E26 oncogene homolog (avian) | 4 | 5.70E-04 | 2.01E-03 |
| CIITA | class II, major histocompatibility complex, transactivator | 4 | 9.77E-04 | 3.27E-03 |
| TWIST1 | twist basic helix-loop-helix transcription factor 1 | 4 | 1.55E-03 | 4.96E-03 |
| STAT6 | signal transducer and activator of transcription 6, interleukin-4 induced | 4 | 1.73E-03 | 5.27E-03 |
| MYB | v-myb myeloblastosis viral oncogene homolog (avian) | 4 | 1.92E-03 | 5.58E-03 |
| NFKBIA | nuclear factor of kappa light polypeptide gene enhancer in B-cells inhibitor, alpha | 3 | 2.42E-03 | 6.75E-03 |
| PPARG | peroxisome proliferator-activated receptor gamma | 5 | 2.62E-03 | 7.01E-03 |
| FOXP2 | forkhead box P2 | 2 | 2.99E-03 | 7.42E-03 |
| ZEB2 | zinc finger E-box binding homeobox 2 | 2 | 2.99E-03 | 7.42E-03 |
| ETV4 | ets variant 4 | 3 | 3.72E-03 | 8.60E-03 |
| REL | v-rel reticuloendotheliosis viral oncogene homolog (avian) | 3 | 3.72E-03 | 8.60E-03 |
| NR3C2 | nuclear receptor subfamily 3, group C, member 2 | 2 | 4.14E-03 | 9.26E-03 |
| TWIST2 | twist basic helix-loop-helix transcription factor 2 | 3 | 5.39E-03 | 1.15E-02 |
| MAZ | MYC-associated zinc finger protein (purine-binding transcription factor) | 2 | 5.47E-03 | 1.15E-02 |
| ETS1 | v-ets erythroblastosis virus E26 oncogene homolog 1 (avian) | 5 | 5.67E-03 | 1.15E-02 |
| RARA | retinoic acid receptor, alpha | 3 | 6.03E-03 | 1.19E-02 |
| SMAD7 | SMAD family member 7 | 2 | 6.97E-03 | 1.33E-02 |
| EPAS1 | endothelial PAS domain protein 1 | 2 | 8.63E-03 | 1.54E-02 |
| PPARD | peroxisome proliferator-activated receptor delta | 2 | 8.63E-03 | 1.54E-02 |
| EGR1 | early growth response 1 | 5 | 8.89E-03 | 1.54E-02 |
| RUNX3 | runt-related transcription factor 3 | 3 | 9.02E-03 | 1.54E-02 |
| FOS | FBJ murine osteosarcoma viral oncogene homolog | 4 | 9.17E-03 | 1.54E-02 |
| CREB1 | cAMP responsive element binding protein 1 | 5 | 9.74E-03 | 1.59E-02 |
| PAX6 | paired box 6 | 2 | 1.05E-02 | 1.63E-02 |
| STAT4 | signal transducer and activator of transcription 4 | 2 | 1.05E-02 | 1.63E-02 |
| ETS2 | v-ets erythroblastosis virus E26 oncogene homolog 2 (avian) | 3 | 1.08E-02 | 1.64E-02 |
| CREB5 | cAMP responsive element binding protein 5 | 2 | 1.24E-02 | 1.85E-02 |
| PPARA | peroxisome proliferator-activated receptor alpha | 3 | 1.85E-02 | 2.69E-02 |
| PTTG1 | pituitary tumor-transforming 1 | 2 | 1.92E-02 | 2.74E-02 |
| RUNX1 | runt-related transcription factor 1 | 3 | 1.98E-02 | 2.76E-02 |
| RFX5 | regulatory factor X, 5 (influences HLA class II expression) | 2 | 2.72E-02 | 3.72E-02 |
| SIRT1 | sirtuin 1 | 3 | 3.18E-02 | 4.26E-02 |

**Supplement table 2** Key transcriptional factors for yellow module.

| Key TF | Description | Targets | P value | FDR |
| --- | --- | --- | --- | --- |
| E2F1 | E2F transcription factor 1 | 23 | 1.38E-25 | 4.98E-24 |
| TP53 | tumor protein p53 | 17 | 2.58E-15 | 4.65E-14 |
| E2F4 | E2F transcription factor 4, p107/p130-binding | 8 | 2.48E-12 | 2.98E-11 |
| YBX1 | Y box binding protein 1 | 8 | 2.84E-11 | 2.56E-10 |
| E2F3 | E2F transcription factor 3 | 6 | 1.97E-10 | 1.42E-09 |
| MYC | v-myc myelocytomatosis viral oncogene homolog (avian) | 9 | 4.09E-08 | 2.46E-07 |
| TFDP1 | transcription factor Dp-1 | 4 | 5.09E-07 | 2.62E-06 |
| ARID3A | AT rich interactive domain 3A (BRIGHT-like) | 3 | 7.05E-06 | 3.17E-05 |
| SP1 | Sp1 transcription factor | 13 | 3.44E-05 | 1.38E-04 |
| EP300 | E1A binding protein p300 | 5 | 4.95E-05 | 1.78E-04 |
| RB1 | retinoblastoma 1 | 4 | 6.78E-05 | 2.22E-04 |
| TP73 | tumor protein p73 | 3 | 9.72E-05 | 2.92E-04 |
| MYCN | v-myc myelocytomatosis viral related oncogene, neuroblastoma derived (avian) | 4 | 2.97E-04 | 8.23E-04 |
| IRF1 | interferon regulatory factor 1 | 4 | 4.82E-04 | 1.13E-03 |
| ATM | ataxia telangiectasia mutated | 3 | 4.99E-04 | 1.13E-03 |
| MED1 | mediator complex subunit 1 | 2 | 5.01E-04 | 1.13E-03 |
| PRDM1 | PR domain containing 1, with ZNF domain | 2 | 7.48E-04 | 1.58E-03 |
| CUX1 | cut-like homeobox 1 | 2 | 1.38E-03 | 2.62E-03 |
| HES1 | hairy and enhancer of split 1, (Drosophila) | 2 | 1.38E-03 | 2.62E-03 |
| HDAC1 | histone deacetylase 1 | 4 | 1.68E-03 | 3.02E-03 |
| MEN1 | multiple endocrine neoplasia I | 2 | 2.68E-03 | 4.59E-03 |
| KLF5 | Kruppel-like factor 5 (intestinal) | 2 | 3.76E-03 | 6.16E-03 |
| NR4A1 | nuclear receptor subfamily 4, group A, member 1 | 2 | 5.02E-03 | 7.23E-03 |
| PTTG1 | pituitary tumor-transforming 1 | 2 | 5.02E-03 | 7.23E-03 |
| TBP | TATA box binding protein | 2 | 5.02E-03 | 7.23E-03 |
| FOXM1 | forkhead box M1 | 2 | 6.44E-03 | 8.92E-03 |
| FOXO3 | forkhead box O3 | 2 | 7.21E-03 | 9.62E-03 |
| CREBBP | CREB binding protein | 2 | 1.37E-02 | 1.76E-02 |
| ESR1 | estrogen receptor 1 | 3 | 1.72E-02 | 2.14E-02 |
| AR | androgen receptor | 3 | 2.91E-02 | 3.50E-02 |
| KLF4 | Kruppel-like factor 4 (gut) | 2 | 3.32E-02 | 3.86E-02 |

**Supplement table 3.** The results of gene set enrichment analysis for HALLMARK in the CGGA and TCGA datasets.

| NAME | CGGA | | TCGA | |
| --- | --- | --- | --- | --- |
|  | NES | p.adjust | NES | p.adjust |
| HALLMARK_INTERFERON_GAMMA_RESPONSE | 3.27 | 2.53E-03 | 3.28 | 2.67E-03 |
| HALLMARK_ALLOGRAFT_REJECTION | 2.99 | 2.53E-03 | 3.06 | 2.67E-03 |
| HALLMARK_INTERFERON_ALPHA_RESPONSE | 2.98 | 2.53E-03 | 2.86 | 2.67E-03 |
| HALLMARK_EPITHELIAL_MESENCHYMAL_TRANSITION | 2.87 | 2.53E-03 | 2.71 | 2.67E-03 |
| HALLMARK_TNFA_SIGNALING_VIA_NFKB | 2.84 | 2.53E-03 | 2.86 | 2.67E-03 |
| HALLMARK_IL6_JAK_STAT3_SIGNALING | 2.83 | 2.53E-03 | 2.69 | 2.67E-03 |
| HALLMARK_INFLAMMATORY_RESPONSE | 2.66 | 2.53E-03 | 2.70 | 2.67E-03 |
| HALLMARK_COAGULATION | 2.60 | 2.53E-03 | 2.48 | 2.67E-03 |
| HALLMARK_HYPOXIA | 2.60 | 2.53E-03 | 2.18 | 2.67E-03 |
| HALLMARK_IL2_STAT5_SIGNALING | 2.56 | 2.53E-03 | 2.37 | 2.67E-03 |
| HALLMARK_COMPLEMENT | 2.55 | 2.53E-03 | 2.54 | 2.67E-03 |
| HALLMARK_GLYCOLYSIS | 2.40 | 2.53E-03 | 2.06 | 2.67E-03 |
| HALLMARK_APOPTOSIS | 2.36 | 2.53E-03 | 2.40 | 2.67E-03 |
| HALLMARK_MTORC1_SIGNALING | 2.30 | 2.53E-03 | 2.13 | 2.67E-03 |
| HALLMARK_REACTIVE_OXYGEN_SPECIES_PATHWAY | 2.29 | 2.53E-03 | 1.86 | 5.28E-03 |
| HALLMARK_P53_PATHWAY | 2.16 | 2.53E-03 | 1.93 | 2.67E-03 |
| HALLMARK_ANGIOGENESIS | 2.14 | 2.53E-03 | 2.23 | 2.85E-03 |
| HALLMARK_KRAS_SIGNALING_UP | 2.06 | 2.53E-03 | 2.20 | 2.67E-03 |
| HALLMARK_DNA_REPAIR | 1.99 | 2.53E-03 | 1.89 | 2.67E-03 |
| HALLMARK_UNFOLDED_PROTEIN_RESPONSE | 1.97 | 2.53E-03 | 1.50 | 1.32E-02 |
| HALLMARK_PI3K_AKT_MTOR_SIGNALING | 1.94 | 2.53E-03 | 1.65 | 2.67E-03 |
| HALLMARK_MYC_TARGETS_V1 | 1.88 | 2.53E-03 | 1.99 | 2.67E-03 |
| HALLMARK_XENOBIOTIC_METABOLISM | 1.84 | 2.53E-03 | 1.61 | 2.67E-03 |
| HALLMARK_APICAL_JUNCTION | 1.80 | 2.53E-03 | 1.51 | 2.67E-03 |
| HALLMARK_UV_RESPONSE_UP | 1.79 | 2.53E-03 | 1.64 | 2.67E-03 |
| HALLMARK_PROTEIN_SECRETION | 1.76 | 2.53E-03 | 1.54 | 1.17E-02 |
| HALLMARK_TGF_BETA_SIGNALING | 1.75 | 2.53E-03 | 1.48 | 3.49E-02 |
| HALLMARK_E2F_TARGETS | 1.73 | 2.53E-03 | 2.31 | 2.67E-03 |
| HALLMARK_ADIPOGENESIS | 1.65 | 2.53E-03 | 1.60 | 2.67E-03 |
| HALLMARK_ESTROGEN_RESPONSE_LATE | 1.52 | 4.08E-03 | 1.53 | 2.67E-03 |
| HALLMARK_G2M_CHECKPOINT | 1.40 | 1.78E-02 | 1.71 | 2.67E-03 |
| HALLMARK_KRAS_SIGNALING_DN | -1.61 | 6.24E-03 | -1.30 | 2.42E-02 |
| HALLMARK_HEDGEHOG_SIGNALING | -1.72 | 8.19E-03 | -1.58 | 3.49E-02 |

**Supplement table 4.** Correlation of risk score with immune cells by CIBERSORT in the CGGA and TCGA datasets.

|  | CGGA | | TCGA | | |
| --- | --- | --- | --- | --- | --- |
|  | R | p value | R | p value |  |
| Macrophages | 0.45 | 0 | 0.63 | 5.48E-70 |  |
| Neutrophils | 0.22 | 5.66E-05 | 0.26 | 6.63E-11 |  |
| Plasma cells | 0.18 | 9.71E-04 | -0.67 | 2.61E-82 |  |
| Eosinophils | 0.15 | 8.01E-03 | -0.02 | 6.85E-01 |  |
| Dendritic cells | 0.11 | 5.95E-02 | 0.13 | 1.86E-03 |  |
| Mast cells | -0.13 | 2.19E-02 | -0.11 | 8.73E-03 |  |
| B cells | -0.21 | 2.14E-04 | -0.39 | 1.85E-24 |  |
| Monocytes | -0.23 | 3.67E-05 | -0.11 | 5.43E-03 |  |
| NK cells | -0.29 | 1.85E-07 | 0.15 | 1.98E-04 |  |
| T cells | -0.31 | 1.02E-08 | 0.22 | 2.15E-08 |  |
